# Discrete Dynamics of Dynamic Neural Fields

**DOI:** 10.3389/fncom.2021.699658

**Published:** 2021-07-08

**Authors:** Eddy Kwessi

**Affiliations:** Department of Mathematics, Trinity University, San Antonio, TX, United States

**Keywords:** dynamic neural fields, discrete, stability, simulations, neurons

## Abstract

Large and small cortexes of the brain are known to contain vast amounts of neurons that interact with one another. They thus form a continuum of active neural networks whose dynamics are yet to be fully understood. One way to model these activities is to use dynamic neural fields which are mathematical models that approximately describe the behavior of these congregations of neurons. These models have been used in neuroinformatics, neuroscience, robotics, and network analysis to understand not only brain functions or brain diseases, but also learning and brain plasticity. In their theoretical forms, they are given as ordinary or partial differential equations with or without diffusion. Many of their mathematical properties are still under-studied. In this paper, we propose to analyze discrete versions dynamic neural fields based on nearly exact discretization schemes techniques. In particular, we will discuss conditions for the stability of nontrivial solutions of these models, based on various types of kernels and corresponding parameters. Monte Carlo simulations are given for illustration.

## 1. Introduction

To model a large amount of cells randomly placed together with a uniform volume density and capable of supporting various simple forms of activity, including plane waves, spherical and circular waves, and vortex effects, Beurle (1956) proposed a continuous model consisting of a partial differential equation, that can capture some neurons behaviors like excitability at the cortical level. Amari (1977) and Wilson and Cowan (1972) subsequent modifications did allow for new features such as inhibition. This essentially gave birth to the theory of dynamic neural fields (DNFs) with far reaching applications. The literature on the DNFs is rather rich, and the trends nowadays are toward cognition, plasticity, and robotics. In cognitive neuroscience for instance, DNFs have enabled analyses of Electroencephalograms (Nunez and Srinivasan, 2006), short term memory (Camperi and Wang, 1998), visual hallucinations (Ermentrout and Cowan, 1979; Tass, 1995), visual memory (Schmidt et al., 2002). Durstewitz et al. (2000) have proposed neurocomputational models of working memory using single layer DNFs and shown that the temporal dynamics they obtained compare well with the *in vivo* observations. Simmering et al. (2008) and Perone and Simmering (2019) used DNFs to achieve spatial cognition by successfully simulating infants' performance in some tasks. Quinton and Goffart (2017) used nonlinearities in DNFs to propose a unifying model for goal directed eye-movements that allow for the implementation of qualitatively different mechanisms including memory formation and sensorimotor coupling. Plasticity in neuroscience can be thought of as the ability of the brain to adapt to external activities by modifying some of its structure. Connections between plasticity and DNFs have been made in studies on intrinsic plasticity, see Strub et al. (2017) or parallel works such as Neumann and Steil (2011), Pozo and Goda (2010), and the references therein. Robotics has been a great niche for DNFs with the works of Bicho et al. (2000), Erlhagen and Schöner (2001), Erlhagen and Bicho (2006), and Bicho et al. (2010).

**Figure 1:**
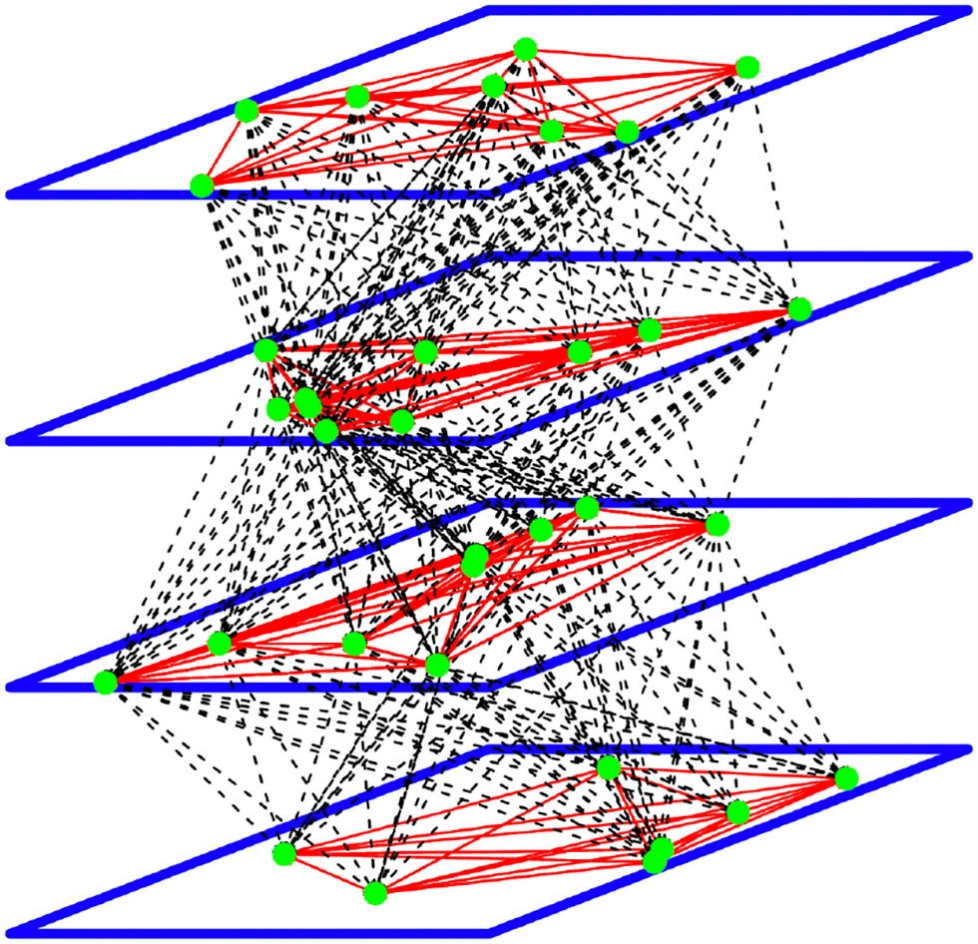
A representation of the DNFs with four layers and 34 neurons. The green dots are the neurons with intra-layer connections represented by the red lines, and inter-layer connections represented by the dashed lines.

To recall the theoretical aspects of DNFs, let Ω ⊆ ℝ^*d*^ be a manifold where *d* is positive integer. In the presence of neurons located on Ω at time *t* arranged on *L* layers, the average potential function *V*_*k*_(*x*_*k*_, *t*) is often used to understand the continuous field on the *k*th layer. *V*_*k*_(*x*_*k*_, *t*) is the average membrane potential of the neuron located at position *x*_*k*_ at time *t* of the *k*th layer. When *L* = 1, *V*(*x*_*k*_, *t*) can also be understood as the synaptic input or activation at time *t* of a neuron at position or direction *x*_*k*_. It satisfies the equation (see Amari, 1977) which is given as

(1) $$\begin{array}{l} \frac{\partial V_{k}\left(x_{k},t\right)}{\partial t}=-V_{k}\left(x_{k},t\right)\\ \;+\overset{L}{\underset{\ell =1}{\sum }}\int_{\Omega }W_{k\ell }\left(x_{k},x_{\ell }\right)G\left(V_{\ell }\left(x_{\ell },t\right)\right)dx_{\ell }+S_{k}\left(x_{k},t\right), \end{array}$$

where *W*_*kl*_(*x*_*k*_, *x*_ℓ_) is the intensity of the connection between a neuron located at position *x*_*k*_ on the *k*th layer with a neuron a position *x*_ℓ_ on the ℓth layer, *G*[*V*_ℓ_(*x*_ℓ_, *t*)] is the pulse emission rate (or activity) at time *t* of the neuron located at position *x*_ℓ_ on the ℓth layer. *G* is often chosen as a monotone increasing function. *S*_*k*_(*x*_*k*_, *t*) represents the intensity of the external stimulus at time *t* arriving on the neuron at position *x*_*k*_ on the *k*th layer, see Figure 1 below.

Most of the mentions of discrete systems involving DNFs are of Euler forward type discretization—see Erlhagen and Bicho (2006), Quinton and Goffart (2017), and Beim and Hutt (2014)—with simulations as the main objective. Stability analysis of solutions of DNFs in the sense of discrete dynamical systems are either understudied or often ignored. Beim and Hutt (2014) studied an heterogeneous aspect of DNFs and found existence of attractors and saddle nodes for solutions of (1). There are also discussion of DNFs as neural networks. For instance, Sussilo and Barack (2013) discussed continuous time recurrent neural network (RNN) as differential equation of the form

(2) $$\begin{array}{l} \frac{\partial V_{k}}{\partial t}=-V_{k}+\overset{M}{\underset{j=1}{\sum }}L_{kj}G\left(V_{j}\right)+\eta_{k}, \end{array}$$

where *V*_*k*_ is an *M* dimensional state of activations and *G*(*V*_*j*_) are the firing rates, and $\eta_{k}=\overset{I}{\underset{j=1}{\sum }}B_{kj}u_{j}$ represents the external stimulus received from *I* inputs *u*_*j*_ and synaptic weights *B*_*kj*_. The weights *L*_*kj*_ are selected from a normal distribution. In a sense, the Equation (1) can be considered a continuous time (recurrent neural networks) RNN. Authors such as Elman (1990) and Williams and Zipser (1990), and most recently, Durstewitz (2017)—see Equation (9.8) therein, have discussed discrete time RNN formulated as a difference equation of the form

(3) $$\begin{array}{l} V_{n+1}^{\left(i\right)}=G\left(W_{i0}+\overset{M}{\underset{j=1}{\sum }}W_{ij}V_{n}^{\left(j\right)}+\eta_{n}^{\left(i\right)}\right). \end{array}$$

where *G* is a monotone increasing function, *W*_*i*0_ is a basic activity level for the neuron at position *x*_*i*_, the *W*_*ij*_'s are weights representing the connection strength between neuron at position *x*_*i*_ and neuron at position *x*_*j*_, and $\eta_{n}^{\left(i\right)}$ is some external stimulus at time *n* arriving on the neuron at position *x*_*i*_. So essentially, after discretization, (1) may yield not only a discrete time dynamical system, but also a RNN. In fact, Jin et al. (2021) considered a discrete equivalent in the space for Equation (1) for *L* = 2 while maintaining the time as continuous and discussed it in the context of unsupervised learning which could help understand plasticity in the brain. The two main goals of this paper are:

1. to propose a discrete dynamic neural field model that is both numerically convergent and dynamically consistent. The model is based on nearly exact discretization techniques proposed in Kwessi et al. (2018) that ensure that the discrete system obtained preserves the dynamics of the continuous system it originated from.
2. to propose a rigorous and holistic theoretical framework to study and understand the stability of dynamic neural fields.

The model we propose has the advantage of being general and simple enough to implement. It also captures different generalizations of DNFs such as predictive neural fields (PNFs) and active neural fields (ANFs). We further show that the proposed model is in effect a Mann-type (see Mann, 1953) local iterative process which admits weak-type solutions under appropriate conditions. The model can also be written as a recurrent neural network. Stability analysis shows that in some case, the model proposed can be studied using graph theory.

The remainder of the paper is organized as follows: in section 2, we propose our nearly exact discretization scheme for DNFs. In section 3.1, we make a brief overview of the essential notions for the stability analysis of discrete dynamical systems, in section 3.2, we discuss the existence on nontrivial fixed solutions for DDNFs, in sections 3.3 and 3.4, we discuss stability analysis for a one and multiple layers discrete DNFs. In section 3.5, we propose simulations and their interpretation for neural activity and in section 4, we make our concluding remarks.

## 2. Materials and Methods

### 2.1. Discrete Schemes for DNFs

For sake of simplification, we will use the following notations: given a positive integer *L*, and integers 1 ≤ *k*, ℓ ≤ *L, M*_*k*_, *M*_ℓ_, we consider indices 1 ≤ *i*_*k*_ ≤ *M*_*k*_, 1 ≤ *j*_ℓ_ ≤ *M*_ℓ_. We put

(4) $$\begin{array}{l} \begin{array}{c} V_{i,n}^{\left(k\right)}:=V_{k}\left(x_{i},t_{n}\right)\\ S_{i,n}^{\left(k\right)}:=S_{k}\left(x_{i},t_{n}\right)\\ \alpha_{n}:=1-\phi \left(h_{n}\right)\\ \beta_{k}:=\frac{\left|\Omega \right|}{M_{k}}\\ W_{ij}^{\left(k,\ell \right)}:=W_{k\ell }\left(x_{i},y_{j}\right) \end{array}, \end{array}$$

where *h*_*n*_ = *t*_*n*+1_ − *t*_*n*_, and $\phi \left(h_{n}\right)=1-e^{-h_{n}}$ is the time scale of the field. We obviously assume that all the layers of the domain Ω have the same time scale. |Ω| represents the size of the field Ω that henceforth we will assume to be finite. *x*_*i*_ represents a point on the *k*th layer and *y*_*j*_ a point on the ℓth layer. *M*_*k*_ represents the number of interconnected neurons on the *k*th layer of the domain Ω, and $\beta_{k}=\frac{\left|\Omega \right|}{M_{k}}$ represents the spatial discretization step on the *k*th layer of the domain Ω. When *L* = 1, we will adopt the notations $V_{i,n}^{\left(1\right)}=V_{i,n},S_{i,n}^{\left(1\right)}:=S_{i,n}$ and $W_{ij}^{\left(1,1\right)}:=W_{ij}$. Henceforth, for sake of clarity and when needed, we will represent vectors or matrices in bold.

To obtain a discrete model for DNFs, we are going to use nearly exact discretization schemes (NEDS) (see Kwessi et al., 2018). We note that according to this reference, Equation (1) is of type *T*_1_, therefore, the left-hand-side will be discretized as

$$\frac{V_{i,n+1}^{\left(k\right)}-V_{i,n}^{\left(k\right)}}{\phi \left(h_{n}\right)}.$$

For the right-hand-side, we write a Riemann sum for the integral in Equation (1), over the domain Ω as

$$\overset{L}{\underset{\ell =1}{\sum }}\frac{\left|\Omega \right|}{M_{\ell }}\overset{M_{\ell }}{\underset{j=1}{\sum }}W_{ij}^{\left(k,\ell \right)}\cdot G\left(V_{j,n}^{\left(\ell \right)}\right)+S_{i,n}^{\left(k\right)}.$$

This means that, for a given *L* ∈ ℕ, for integers 1 ≤ *k* ≤ *L, M*_*k*_, and 1 ≤ *i*_*k*_ ≤ *M*_*k*_, a discrete dynamic neural field (DDNFs) model can be written as

(5) $$\begin{array}{l} V_{i,n+1}^{\left(k\right)}=\alpha_{n}V_{i,n}^{\left(k\right)}+\left(1-\alpha_{n}\right)\overset{L}{\underset{\ell =1}{\sum }}\overset{M_{\ell }}{\underset{j=1}{\sum }}\beta_{\ell }\cdot W_{ij}^{\left(k,\ell \right)}\cdot G\left(V_{j,n}^{\left(\ell \right)}\right)\\ \;+\left(1-\alpha_{n}\right)S_{i,n}^{\left(k\right)}. \end{array}$$

Staying within the confines of the layers' architecture proposed by Amari (1977), in a DDNFs with *L* ≥ 3, we observe that the activity on neurons located on the bottom (or top) layer depends on the interconnected neurons on that layer and their connections to the layer above (or below). First, we define

$$L_{k}=\{\begin{array}{ll} \left\{1,2\right\} & \text{if }k=1\\ \left\{k-1,k,k+1\right\} & \text{if }1<k<L\\ \left\{L-1,L\right\} & \text{if }k=L \end{array}.$$

Middle layers are connected to two layers, one above and one below. This means that at time *t*_*n*_, a neuron at position *x*_*i*_ on the first layer receives external input $S_{i,n}^{\left(1\right)}$, intra-layer signals $W_{ij}^{\left(1,1\right)}$ from *M*_1_ neurons on the first layer, and inter-layer signals $W_{ij}^{\left(1,2\right)}$ from *M*_2_ neurons on the second layer. Likewise, a neuron at position *x*_*i*_ on the *L*th layer receives external input $S_{i,n}^{\left(L\right)}$, inter-layer signals $W_{ij}^{\left(L-1,L\right)}$ from *M*_*L*−1_ neurons on the (*L* − 1)th layer, and intra-layer signals $W_{ij}^{\left(L,L\right)}$ from *M*_*L*_ neurons on the *L*th layer. For 1 < *k* < *L*, we have $W_{ij}^{\left(k,\ell \right)}=0$ if ℓ ∉ *L*_*k*_ and $W_{ij}^{\left(k,\ell \right)}\neq 0$ if ℓ ∈ *L*_*k*_. This means that at time *t* = *t*_*n*_, a neuron at position *x*_*i*_ on the *k*th layer receives external input $S_{i,n}^{\left(k\right)}$, inter-layer signals $W_{ij}^{\left(k-1,k\right)}$ from *M*_*k*−1_ neurons on the (*k* − 1)th layer, intra-layer signals $W_{ij}^{\left(k,k\right)}$ from *M*_*k*_ neurons on the *k*th layer, and inter-layer signals $W_{ij}^{\left(k,k+1\right)}$ from *M*_*k*+1_ neurons on the (*k* + 1)th layer.

Further, Equation (5) has a matrix notation which helps with the study of stability analysis. Indeed, let $M=\underset{1\leq k\leq L}{\text{max}}M_{k}$ and *d* = *L* × *M*, let ***X* = (*X*_1_, *X*_2_, ⋯ , *X*_*L*_)** ∈ Ω, where for 1 ≤ *k* ≤ *L*, ***X*_*k*_** = {(*x*_*k*1_, *x*_*k*2_, ⋯ , *x*_*kM*_)} represents the positions of *M*_*k*_ neurons on the *k*th layer. We are assuming without loss of generality that *x*_*ki*_ = 0 if *M*_*k*_ < *i* ≤ *M* to have a full *L* × *M* matrix. Now consider the matrix

$$V_{n}\left(X\right)=\left[V_{n}^{\left(1\right)}\left(X\right),V_{n}^{\left(2\right)}\left(X\right),\cdots \;,V_{n}^{\left(L\right)}\left(X\right)\right],$$

formed by the column vectors $V_{n}^{\left(k\right)}\left(X\right)={\left(V_{1,n}^{\left(k\right)},V_{2,n}^{\left(k\right)},\cdots \;,V_{M,n}^{\left(k\right)}\right)}^{T}$. In this case, Equation (5) can be written in a matrix form as

(6) $$\begin{array}{l} V_{n+1}\left(X\right)=F\left(V_{n}\left(X\right)\right)=\left[f_{1}\left(V_{n}\left(X\right)\right),f_{2}\left(V_{n}\left(X\right)\right),\cdots \;,f_{L}\left(V_{n}\left(X\right)\right)\right], \end{array}$$

with an element-wise notation

$$\begin{array}{l} {\left[f_{k}\left(V_{n}\left(X\right)\right)\right]}_{i}=\alpha_{n}V_{i,n}^{\left(k\right)}+\left(1-\alpha_{n}\right)\underset{\ell \in L_{k}}{\sum }\overset{M_{\ell }}{\underset{j=1}{\sum }}\beta_{\ell }\cdot W_{ij}^{\left(k,\ell \right)}\cdot G\left(V_{j,n}^{\left(\ell \right)}\right)\\ \;+\left(1-\alpha_{n}\right)S_{i,n}^{\left(k\right)}, \end{array}$$

for 1 ≤ *i* ≤ *M* and for 1 ≤ *k* ≤ *L*. For more complex layers' architectures, the interested reader can refer to De Domenico et al. (2013) and the references therein, where tensor products are used instead of matrices.

### 2.2. Pulse Emission Rate Functions

**Figure 2:**
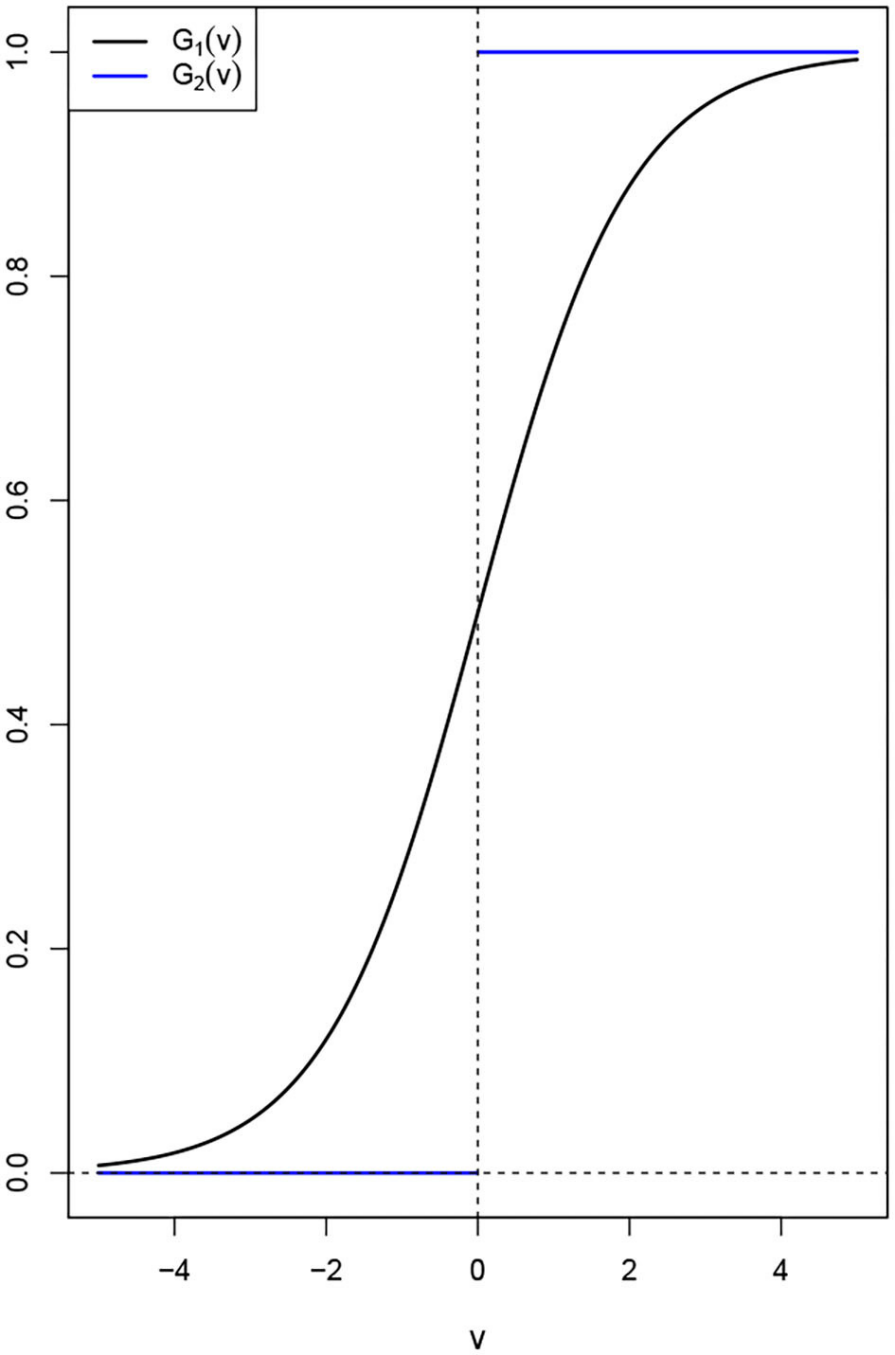
An illustration of the pulse emission rate functions *G*_1_(*v*) and *G*_2_(*v*) for *v*_0_ = 0.

Amari (1977) proved the existence of nontrivial solutions for DNFs when *G* is the Heaviside function. Further studies have shown that in fact this is true for a large classes of functions *G*. As we stated earlier, functions *G* satisfying the Hammerstein conditions *G*(*v*) ≤ μ_1_*v* + μ_2_ are good candidates. However most practitioners use either the Heaviside function or the sigmoid function. For some θ > 0 and *v*_0_ ≥ 0, the sigmoid function, is defined as $G_{1}\left(v,v_{0}\right)={\left(1+e^{-\theta \left(v-v_{0}\right)}\right)}^{-1}$, and Heaviside function is defined as *G*_2_(*v, v*_0_) = *I*_(_*v*__0_, ∞)_(*v*), see Figure 2 below. Here *I*_*A*_ is the indicator function and is given as *I*_*A*_(*x*) = 1 if *x* ∈ *A* and *I*_*A*_(*x*) = 0 otherwise. This shows that the assumption that *G* be nonnegative and increasing is not unrealistic. In fact we can transform many functions to bear similar properties of the Heaviside and sigmoid functions, see for instance Kwessi and Edwards (2020).

**Remark 1**. *We observe that the choice of the sigmoid activation function* $G_{1}\left(v\right)={\left(1+e^{-\theta \left(v-v_{0}\right)}\right)}^{-1}$ *is widely preferred in the literature for its bounded nature. Another reason is the fact that it is also suitable when the* $X_{n}^{\left(i\right)}$ *are binary, that is, they may take values 0, 1, where 0 and 1 represents, respectively active and non active neurons at time n. In this case*, $G_{1}\left(W_{i0}+\overset{M}{\underset{j=1}{\sum }}W_{ij}X_{n}^{\left(j\right)}+\eta_{n}^{\left(i\right)}\right)=Pr\left(X_{n+1}^{\left(i\right)}=1\right)$ *would represent the probability that there is an activity on neuron at position x*_*i*_ *at time n* + 1.

### 2.3. Connection Intensity Functions

There are a variety of connection intensities functions (or kernels) that one can choose from. This include a Gaussian kernel $Gau\left(x,y,\sigma \right)=\frac{1}{\sigma \sqrt{2\pi }}e^{-\frac{1}{2\sigma^{2}}∥x-y{∥}^{2}}$, the Laplacian kernel defined as $Lap\left(x,y,\sigma \right)=\frac{1}{2\sigma }e^{-\frac{1}{\sigma }∥x-y∥}$, or the hyperbolic tangent kernel *Thyp*(*x, y*, β, *r*) = 1 + tanh(β*x*^*T*^ · *y* + *r*) where ∥·∥ is a norm in ℝ^*d*^. Note that as defined, the hyperbolic tangent kernel is not symmetric, however, for a suitable choice of β and *r*, one may obtain a nearly symmetric kernel. Indeed, if *r* < 0 and β is close to zero, we have *Thyp*(*x, y*, β, *r*) ≈ (1 + tanh(*r*))*e*^−β∥*x*−*y*∥^2^^, see for instance Lin and Lin (2003). This kernel is very useful in non-convex problems and support vector machines.

**Figure 3:**
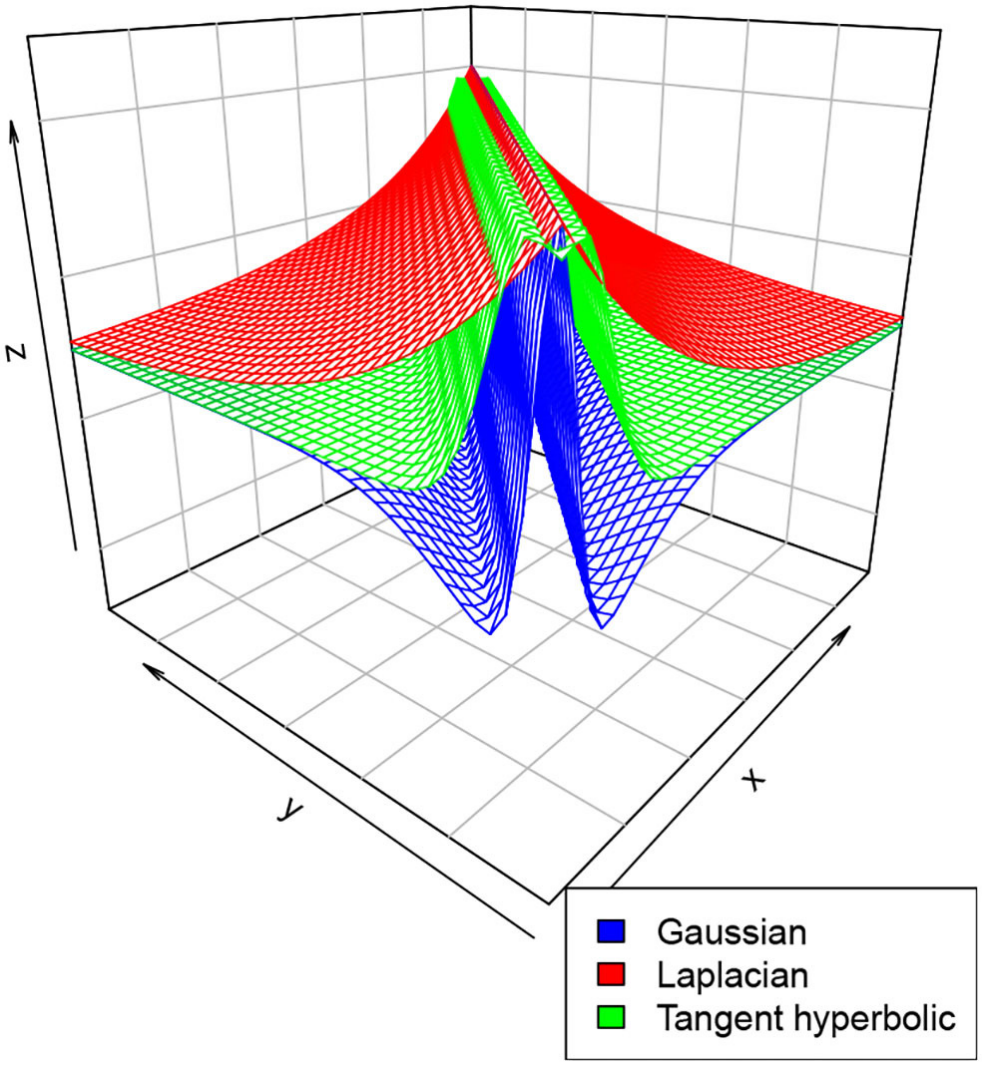
An illustration of the connection intensity functions defined above for $\sigma^{+}=4.5,\sigma^{-}=1.5,\sigma_{1}=1,\sigma_{2}=4.5,\beta_{1}=0.1,\beta_{2}=0.5$, and *r* = − 0.1.

In practice however, it is very common to select the function *W*(*x, y*) as the difference between either of the functions above, which results in a “Mexican hat” shaped function, that is, either $W\left(x,y\right)=\sigma^{+}Gau\left(x,y,\sigma_{1}\right)-\sigma^{-}Gau\left(x,y,\sigma_{2}\right)$, or $W\left(x,y\right)=\sigma^{+}Lap\left(x,y,\sigma_{1}\right)-\sigma^{-}Lap\left(x,y,\sigma_{2}\right)$, or $W\left(x,y\right)=\sigma^{+}Thyp\left(x,y,\beta_{1},r\right)-\sigma^{-}Thyp\left(x,y,\beta_{2},r\right)$. Figure 3 below is an illustration of these kernels for selected parameters. This choice will ensure that neurons within the same cell assembly are connected reciprocally by high synaptic weights σ^+^, whereas neurons not belonging to the same assembly are connected by low synaptic weights σ^−^ (see for instance Durstewitz et al., 2000; Quinton and Goffart, 2017; Wijeakumar et al., 2017; Jin et al., 2021).

## 3. Existence and Stability of Nontrivial Solutions of the DDNFs

### 3.1. Stability Analysis

We recall the essential notions for the stability analysis of a discrete dynamical system *V*_*n*+1_ = *F*(*V*_*n*_) where *F* : ℝ^*d*^ → ℝ is continuously differentiable map.

**Definition 2**.

- *A point V*_∗_ *is said to be a fixed point for F if F*(*V*_∗_) = *V*_∗_.
- *The orbit* O(V) *of a point V is defined as* $O\left(V\right)=\left\{F^{n}\left(V\right),\text{where}\;n\in {\mathbb{Z}}_{+}\right\}$ *with* $F^{n}=\underset{n\;times}{\underset{︸}{F◦F◦\cdots ◦F}}$.
- *The spectral radius* ρ(*A*) *a matrix A is a maximum of its absolute eigenvalues*.
- *A fixed point V*_∗_ *for F is said to be stable if there exists a neighborhood of V*_∗_ *whose trajectories are arbitrarily close to V*_∗_, *that is, for all* ϵ > 0, $\exists \delta >0:|V-V_{*}|<\delta \Rightarrow |F^{n}\left(V\right)-V_{*}|<\epsilon$.
- *A fixed point V*_∗_ *for F is said to be attracting if there exists a neighborhood of V*_∗_ *whose trajectories converge to V*_∗_, *that is, for all* ϵ > 0, $\exists \delta >0:|V-V_{*}|<\delta \Rightarrow \underset{n\to \infty }{\text{lim}}F^{n}\left(V\right)=V_{*}$.
- *A fixed point V*_∗_ *for F is said to be asymptotically stable if it is both stable and attracting*.
- *A fixed point V*_∗_ *for F is said to be unstable if it is not stable*.

**Theorem 3**. *Let V*_*n*+1_ = *F*(*V*_*n*_) *be a discrete dynamical system with a fixed point V*_∗_ *and let A* = *JF*(*V*_∗_) *be its Jacobian matrix*.

1. *If* ρ(*A*) < 1, *then V*_∗_ *is asymptotically stable*.
2. *If* ρ(*A*) > 1, *then V*_∗_ *is unstable*.
3. *If* ρ(*A*) = 1, *then V*_∗_ *may be stable or unstable*.

### 3.2. Existence of Nontrivial Fixed Solutions for the DDNFs Model

A nontrivial solution for the DNFs is a non constant solution for which the left-hand side of Equation (1) is equal to zero. For the DDNFs, it can be formulated in the following way: for a given *x*_*i*_ on the *k*th layer, the DDNFs in (5) has a nontrivial fixed point $V_{i,*}^{\left(k\right)}$ if for all *n* ≥ 1, we have $V_{i,n+1}^{\left(k\right)}=F\left(V_{i,n}^{\left(k\right)}\right)=V_{i,n}^{\left(k\right)}:=V_{i,*}^{\left(k\right)}$. This is satisfied when

(7) $$\begin{array}{l} V_{i,n}^{\left(k\right)}=\underset{\ell \in L_{k}}{\sum }\overset{M_{\ell }}{\underset{j=1}{\sum }}\beta_{\ell }\cdot W_{i,j}^{\left(k,\ell \right)}\cdot G\left(V_{j,n}^{\left(\ell \right)}\right)+S_{i}^{\left(k\right)}. \end{array}$$

The right-hand side of Equation (7) is a Riemann sum for

$$\overset{L}{\underset{\ell =1}{\sum }}\int_{\Omega }W_{k\ell }\left(x,y\right)G\left(V_{\ell }\left(y,t\right)\right)dy+S_{k}\left(x\right).$$

So the existence of a nontrivial fixed point for the continuous DNFs Equation (1) implies the existence of a non trivial fixed solution for the DDNFs. According to Hammerstein (1930), such a nontrivial solution exists if *W*(*x, y*) is symmetric positive definite and *G* satisfies *G*(*v*) ≤ μ_1_*v* + μ_2_, where 0 < μ_1_ < 1, μ_2_ > 0 are constants.

We will use a different approach to prove existence of nontrivial solution of DNFs. Let (S,A,μ) be a measure space. We will consider the space *L*^1^(*S*) of equivalent classes of integrable functions on *S*, endowed with the norm $∥f∥=\int_{S}|f\left(x\right)|dx$, where *dx* = μ(*dx*).

**Definition 4**. *A stochastic kernel W is a measurable function W* : *S* × *S* → ℝ *such that*

1. *W*(*x, y*) ≥ 0, ∀*x, y* ∈ *S*,
2. $\int_{S}W\left(x,y\right)dy=1,\forall x\in S$.

**Definition 5**. *A density function f is an element of L*^1^(*S*) such that *f* : *S* → ℝ such that $\int_{S}fd\mu =1$.

**Definition 6**. *A Markov operator P on L*^1^(*S*) *is a positive linear operator that preserves the norm, that is, P* : *L*^1^(*S*) → *L*^1^(*S*) *is linear such that*

1. *Pf* ≥ 0 *if f* ≥ 0 and *f* ∈ *L*^1^(*S*),
2. ∥*Pf*∥ = ∥*f*∥.

**Remark 7**.

*We note that f* ≥ 0 *(and Pf* ≥ 0*) represents equivalent classes of integrable functions on S that are greater or equal to 0 except on a set of measure zero and thus the inequality should be understood almost everywhere*.

*We also note that when an operator *P* satisfies the second condition above, it is sometimes referred to as a non-expansive operator*.

**Theorem 8**. *(Corollary 5.2.1–Lasota and Mackey, 2004). Let* S≡(S,A,μ) *be measure space and P* : *L*^1^(*S*) → *L*^1^(*S*) *be a Markov operator. If for some density function f, there is g* ∈ *L*^1^(*S*) *such that P*^*n*^*f* ≤ *g*, *for all n, then*

$$there\;is\;a\;density\;f_{*}\;such\;that\;Pf_{*}=f_{*}.$$

Now we use Definitions 4, 5, and 6 and Theorem 8 above to state our result on the existence of nontrivial fixed solution of DNFs.

**Theorem 9**. *Let* $S:=\Omega =\prod_{i=1}^{d}\left[a_{i},b_{i}\right]\subset {\mathbb{R}}^{d}$. *Consider a DNFs with no external stimulus [S*(*x*) = 0*]. If W*(*x, y*) *is positive and bounded, then the DNFs (and consequently the DDNFs) has at least one nontrivial fixed solution V*_∗_.

Proof. Let A=B(S) be the Borel σ-algebra on *S* generated by the family $\left\{\prod_{i=1}^{d}\left(l_{i},u_{i}\right):a_{i}<l_{i}<u_{i}<b_{i}\right\}$ and μ be the associated Borel measure. A nontrivial solution *V*(*x*) for the DNFs satisfies the equation

$$V\left(x\right)=\int_{S}W\left(x,y\right)G\left(V\left(y\right)\right)dy,\;\text{for all }x\in S.$$

Consider the linear operator *P* defined on *S* as

$$Pf\left(x\right)=\int_{S}W\left(x,y\right)f\left(y\right)dy,\;\text{where }f\left(x\right)=G\left(V\left(x\right)\right).$$

Clearly, *P* is a Markov operator and we have

$$\forall n\geq 1,\;P^{n}f\left(x\right)=\int_{S}W_{n}\left(x,y\right)f\left(y\right)dy,$$

with *W*_1_(*x, y*) = *W*(*x, y*) and $W_{n+1}\left(x,y\right)=\int_{S}W\left(x,z\right)W_{n}\left(z,y\right)dz$ for *n* ≥ 1. Moreover, by the positivity and boundedness of *W*, there exists *M* > 0 such that for a density function *f*, we have *P*^*n*^*f*(*x*) ≤ *M*, ∀*n* ≥ 1. By the above Theorem, there exists a density *f*_∗_ such that *Pf*_∗_ = *f*_∗_. We conclude by observing that *f*_∗_(*x*) = *G*(*V*_∗_(*x*)) = *V*_∗_(*x*) for some function *V*_∗_(*x*) defined on Ω and thus a nontrivial solution for the DNFs and DDNFs exists. That is, *V*_∗_ is a fixed point of *G*.

One major limitation of the above theorem is that it only applies to positive connectivity functions *W*(*x, y*) which would exclude a wide range of DNFs, for example, competing DNFs use almost exclusively negative weight functions as well as DNFs for working memories, see for instance Zibner et al. (2011). One remedy could be to prove the existence of weak fixed solutions of (5) using iterative processes without a positivity restriction. Indeed, let Ω be a nonempty, closed and convex subset of *l*^2^, the space of infinite sequences ***v*** = (*v*_1_, *v*_2_, ⋯ ) such that $∥v{∥}_{2}^{2}=\overset{\infty }{\underset{n=1}{\sum }}|v_{n}{|}^{2}<\infty$. We recall that *l*^2^ is a Hilbert space with the inner product $\langle v,w\rangle =\overset{\infty }{\underset{n=1}{\sum }}v_{n}\bar{w_{n}}$.

**Definition 10**. *Consider a map T* : Ω → *l*^2^. *Suppose there is constant C such that* ∥*T*(***v*_1_**) − *T*(***v*_2_**)∥_2_ ≤ *C*∥***v*_1_ − *v*_2_**∥_2_. *Then*

1. *T is called a Lipschitz map if C* > 0.
2. *T is called a contraction map if 0 < C* < 1.
3. *T is called a non-expansive map if C* = 1.

**Definition 11**. *Let T* : Ω → *l*^2^ *be either a contraction or a non-expansive map. A Mann-type iterative process is a difference equation on* Ω *defined as*

(8) $$\begin{array}{l} \{\begin{array}{ll} V_{0} & \in \Omega \\ V_{n+1} & =\alpha_{n}V_{n}+\left(1-\alpha_{n}\right)T\left(V_{n}\right) \end{array}. \end{array}$$

**Theorem 12**. *Suppose* Ω *is a subset of l*^2^ *and W*(*x, y*) *is bounded by W*_0_. *If G*(*v*) *is Lipschitz with Lipschitz constant K such that W*_0_*C* ≤ 1, *then the DDNFs (5) is a Mann-type iterative process with at least one weak solution*.

Proof. Here, we denote *V*_*n*_ to mean *V*_*n*_(***X***) introduced earlier. We start by rewriting certain quantities in vector and matrix form. Let *d* = *L* × *M* where as above, $M=\underset{1\leq k\leq L}{\text{max}}M_{k}$ and let $S_{n}=\left\{S_{i,n}^{k},1\leq k\leq L,1\leq i\leq M\right\}$. Let $G\left(V_{n}^{\left(\ell \right)}\right)={\left(G\left(V_{j,n}^{\left(\ell \right)}\right)\right)}_{1\leq j\leq M}$ and $\Gamma_{i}^{\left(k,\ell \right)}={\left(\beta_{\ell }W_{i,j}^{\left(k,\ell \right)}\right)}_{1\leq j\leq M}$, where as above, we complete the vectors with zeros for *M*_ℓ_<*j* ≤ *M*. Then we have the dot product

$$\Gamma_{i}^{\left(k,\ell \right)}\cdot G\left(V_{n}^{\left(\ell \right)}\right)=\overset{M_{\ell }}{\underset{j=1}{\sum }}\beta_{\ell }W_{ij}^{\left(k,\ell \right)}G\left(V_{j,n}^{\left(\ell \right)}\right),\;1\leq i\leq M,\ell \in L_{k}.$$

Similarly, let $\Gamma_{i}^{\left(k\right)}={\left(\Gamma_{i}^{\left(k,\ell \right)}\right)}_{\ell \in L_{k}}$ and $G\left(V_{n}\right)={\left(G\left(V_{n}^{\left(\ell \right)}\right)\right)}_{\ell \in L_{k}}$. We also have the dot product

$$\Gamma_{i}^{\left(k\right)}\cdot G\left(V_{n}\right)=\underset{\ell \in L_{k}}{\sum }\Gamma_{i}^{\left(k,\ell \right)}\cdot G\left(V_{n}^{\left(\ell \right)}\right),\;1\leq i\leq M.$$

Now we consider the matrix $\Gamma ={\left[\Gamma^{\left(1\right)},\Gamma^{\left(2\right)},\cdots ,\Gamma^{\left(L\right)}\right]}^{T}$ formed by block matrices $\Gamma^{\left(k\right)}=\left(\Gamma_{1}^{\left(k\right)},\Gamma_{2}^{\left(k\right)},\cdots \;,\Gamma_{M}^{\left(k\right)}\right)$ for 1 ≤ *k* ≤ *L*. We observe that (5) is a Mann-type process written as

(9) $$\begin{array}{l} \{\begin{array}{l} V_{0}\in \Omega \\ V_{n+1}=\alpha_{n}V_{n}+\left(1-\alpha_{n}\right)T\left(V_{n}\right) \end{array}, \end{array}$$

where *T* is the linear map on Ω defined as *T*(*V*_*n*_) = Γ · *G*(*V*_*n*_)+*S*_*n*_. It follows that for *U*_*n*_, *V*_*n*_ ∈ Ω

$$\begin{array}{l} ∥T\left(V_{n}\right)-T\left(U_{n}\right){∥}_{2}=∥\Gamma \left[G\left(U_{n}\right)-G\left(V_{n}\right)\right]{∥}_{2}\\ \;\leq W_{0}∥G\left(U_{n}\right)-G\left(V_{n}\right){∥}_{2}\\ \;\leq CW_{0}∥U_{n}-V_{n}{∥}_{2},\;\text{since }G\;\text{is Lipschitz}. \end{array}$$

Since by hypothesis *CW*_0_ ≤ 1, *T* is either a contraction or a non-expansive map. It is known (see Mann, 1953) that in this case, the Mann-type iterative process (9) has at least one weak solution *V*_∗_, that is, there exists *V*_∗_ ∈ Ω such that $\lim_{n\to \infty }\langle V_{n},V\rangle =\langle V_{*},V\rangle$ for all *V* ∈ *l*^2^.

The second equation in (9) is a generalization of Equation (7) in Quinton and Goffart (2017) and according to their description, it can be considered a predictive neural field (PNF) where *T*(*V*_*n*_) is an internal projection corresponding to the activity required to cancel the lag of the neural field *V*_*n*_ for a specific hypothetically observed trajectory, by exciting the field in the direction of the motion, ahead of the current peak location, and inhibiting the field behind the peak.

### 3.3. Stability Analysis of Hyperbolic Fixed Solutions

To discuss stability analysis for the DDNFs in (5), we are going to separate the analysis into a single-layer system and a multiple-layers system. For simplification purposes, we consider the following notations when necessary:

$$\begin{array}{l} K_{ij}^{\left(k,\ell \right)}:=W_{ij}^{\left(k,\ell \right)}G^{\prime }\left(V_{j,*}^{\left(\ell \right)}\right),\;{\bar{K}}_{i,\cdot }^{\left(k,\ell \right)}:=\frac{1}{M_{\ell }}\overset{M_{\ell }}{\underset{j=1}{\sum }}K_{ij}^{\left(k,\ell \right)}. \end{array}$$

If *L* = 1 and for any *M*_1_ > 0, we will write $K_{ij}:=K_{ij}^{\left(1,1\right)}$ and ${\bar{K}}_{i,\cdot }:={\bar{K}}_{i,\cdot }^{\left(1,1\right)}$.

If *L* > 1 and *M*_*k*_ = *M*_ℓ_ = 1 for all 1 ≤ *k*, ℓ ≤ *L*, we will write $K^{\left(k,\ell \right)}:=K_{11}^{\left(k,\ell \right)}$.

#### 3.3.1. Single-Layer DDNFs Model

Let *x*_*i*_ ∈ Ω. Suppose that there are *M* neurons interconnected and connected to the neuron located at *x*_*i*_. Let *W*_*ij*_ be the intensity of the connection between the neuron at *x*_*i*_ and the neuron at *x*_*j*_. Let

$$\mu_{i}=\frac{\left|\Omega \right|}{M}\overset{M}{\underset{j=1}{\sum }}W_{ij}G^{\prime }\left(V_{j,*}\right)=|\Omega |{\bar{K}}_{i,\cdot }$$

be the weighted average rate of change of pulse emissions received or emitted by the neuron at *x*_*i*_, where $V_{i,*}:=V_{i,*}^{\left(1\right)}$ is a nontrivial solution of Equation (5) with *L* = 1. Some authors like Lin et al. (2009) specify the type of connection, by dividing the connectivities or synaptic strengths *W*_*ij*_ into a group of receptive signals called “feed backward” and a group of emission signals called “feed forward.” Here we make no such specification. Recall that $\alpha_{n}=1-\phi \left(h_{n}\right)=e^{-h_{n}}$. Henceforth, we fix *h*_*n*_ = *h* > 0 for all *n* so that $0<\alpha :=\alpha_{n}=e^{-h}<1$.

**Theorem 13**. *Let* Ω ⊆ ℝ *be a bounded region. Let M be a positive integer. Let **x*** = (*x*_*i*_)_1≤*i*≤*M*_ *be a vector of M interconnected points on* Ω *via a connectivity function W*(·, ·).

*Let **V***_**∗**_**(*****x*****)** = (*V*_*i*,∗_)_1≤*i*≤*M*_ *be a nontrivial fixed point of the DDNFs on* Ω.

$$\text{If }\underset{1\leq i\leq M}{\max}|\mu_{i}|<1,then\;V_{*}\left(x\right)\;is\;asymptotically\;stable.$$

Proof. We have $\beta_{l}=\beta =\frac{\left|\Omega \right|}{M}$. Let *x*_*i*_ ∈ Ω and let *V*_*i*,∗_ be a nontrivial fixed point of the DDNFs with one layer. Let 1 ≤ *j* ≤ *M* and *x*_*j*_ ∈ Ω. The DDNFs with one layer in Equation (5) can be written as

$$V_{n+1}\left(x\right)=f\left(V_{n}\left(x\right)\right)=\left(V_{1,n+1},V_{2,n+1},\cdots \;,V_{M,n+1}\right),$$

and element-wise as

$$V_{i,n+1}=\alpha V_{i,n}+\left(1-\alpha \right)\frac{\left|\Omega \right|}{M}\overset{M}{\underset{j=1}{\sum }}W_{ij}G\left(V_{j,n}\right)+S_{i,n}.$$

The function *f* : ℝ^*M*^ → ℝ is a continuously differentiable function since the function *G* : ℝ → ℝ is, and given 1 ≤ *i, p* ≤ *M*, we have that

$$\begin{array}{l} \frac{\partial f({V_{n}\left(x\right))}_{i}}{\partial V_{p,n}}=\frac{\partial V_{i,n+1}}{\partial V_{p,n}}=\alpha \delta_{ip}+\left(1-\alpha \right)\frac{\left|\Omega \right|}{M}\overset{M}{\underset{j=1}{\sum }}W_{ij}G^{\prime }\left(V_{j,n}\right)\delta_{jp}\\ \;=\alpha \delta_{ip}+\left(1-\alpha \right)\frac{\left|\Omega \right|}{M}W_{ip}G^{\prime }\left(V_{p,n}\right), \end{array}$$

where δ_*ij*_ is the Kronecker symbol with δ_*ij*_ = 1 if *i* = *j* and δ_*ij*_ = 0 if *i* ≠ *j*. It follows that

$$\frac{\partial f({V_{n}\left(x\right))}_{i}}{\partial V_{p,n}}=\{\begin{array}{ll} \alpha +\left(1-\alpha \right)\frac{\left|\Omega \right|}{M}W_{ii}G^{\prime }\left(V_{i,n}\right) & \text{when }p=i\\ \left(1-\alpha \right)\frac{\left|\Omega \right|}{M}W_{ip}G^{\prime }\left(V_{p,n}\right) & \text{when }p\neq i \end{array}.$$

We define the adjacency matrix as the Jacobian matrix *A* = *Jf*[***V*_∗_(*x*)**] where *A* = (*a*_*ij*_) with $a_{ij}=\frac{\partial f({V_{*}\left(x\right))}_{i}}{\partial V_{j,n}}$. *A* is a *M* × *M* matrix and let ρ(*A*) be its spectral radius. Therefore, we have

$$\begin{array}{l} \rho \left(A\right)\leq ∥A{∥}_{\infty }=\underset{1\leq i\leq M}{\max}\overset{M}{\underset{j=1}{\sum }}|a_{ij}|\\ \;\leq \underset{1\leq i\leq M}{\max}\overset{M}{\underset{j=1}{\sum }}\left[\alpha \delta_{ij}+\left(1-\alpha \right)|\frac{\left|\Omega \right|}{M}W_{ij}G^{\prime }\left(V_{j,*}\right)|\right]\\ \;=\underset{1\leq i\leq M}{\max}\left[\alpha +\left(1-\alpha \right)|\frac{\left|\Omega \right|}{M}\overset{M}{\underset{j=1}{\sum }}W_{ij}G^{\prime }\left(V_{j,*}\right)|\right]\\ \;=\underset{1\leq i\leq M}{\max}\left[\alpha +\left(1-\alpha \right)|\mu_{i}|\right]\\ \;=\left[\alpha +\left(1-\alpha \right)\underset{1\leq i\leq M}{\max}|\mu_{i}|\right]. \end{array}$$

Since 0 < α < 1, the last equality is equivalent to

$$\min\left\{1,\underset{1\leq i\leq M}{\max}|\mu_{i}|\right\}<∥A{∥}_{\infty }<\max\left\{1,\underset{1\leq i\leq M}{\max}|\mu_{i}|\right\}.$$

It follows that if $\underset{1\leq i\leq M}{\text{max}}|\mu_{i}|<1$, then we have that $\rho \left(A\right)\leq ∥A{∥}_{\infty }<\max\left\{1,\underset{1\leq i\leq N}{\text{max}}|\mu_{i}|\right\}=1$ and consequently, ***V*_∗_(*x*)** is asymptotically stable.

**Remark 14**.

*The condition* $\underset{1\leq i\leq M}{\text{max}}\mu_{i}<1$ *links the size of the domain* Ω *and other parameters such as the rate of change of the pulse emission function G, the connectivity function W*(*x, y*) *and the number of connected neurons M. Indeed*, $\mu_{i}=|\Omega |{\bar{K}}_{i,\cdot }$. *Therefore, achieving* $\underset{1\leq i\leq M}{\text{max}}\mu_{i}<1$ *requires* $\underset{1\leq i\leq N}{\text{max}}{\bar{K}}_{i\cdot }<\frac{1}{\left|\Omega \right|}$. *This means that to ensure stability of the fixed solution **V_∗_*****(*****x*****)**, *it is enough to have weighted rates of change of pulse emission* $K_{ij}=W_{ij}G^{\prime }\left(V_{*}\left(x_{j}\right)\right)$ *be bounded by 1 for each neuron. This condition is obviously true if the neuron at x*_*i*_ *is not connected with the neuron at x*_*j*_ *since W*_*ij*_ *would be zero. In fact the latter corresponds to the trivial fixed solution **V_∗_*****(*****x*****)** = 0.

#### 3.3.2. Special Case of Single Layer Model With a Heaviside Pulse Emission Function

Suppose the pulse emission function *G*(*x*) is the Heaviside function *G*_2_(*v, v*_0_). There are two possible approaches to discuss the stability of the nontrivial solution. First, we observe that the derivative $G_{2}^{\prime }\left(v,v_{0}\right)$ of *G*_2_(*v, v*_0_) exists in the sense of distribution and is given as $G_{2}^{\prime }\left(v,v_{0}\right)=\delta \left(v_{0}\right)$ where δ(*v*_0_) is the Dirac measure at *v*_0_. It can be written as $G_{2}^{\prime }\left(v,v_{0}\right)=0$ if *v* ≠ *v*_0_ and $G_{2}^{\prime }\left(v,v_{0}\right)=\infty$ if *v* = *v*_0_. This means that for a nontrivial solution, the adjacency matrix *A* defined above is reduced to the diagonal matrix *A* = diag(α). It follows that ρ(*A*) = α < 1 and the nontrivial solution *V*_∗_(*x*) is asymptotically stable. Another approach is to note that Equation (5) with one layer (*L* = 1) can be written as

(10) $$\begin{array}{l} V_{i,n+1}=\alpha V_{i,n}+Y_{i,n+1},\;1\leq i\leq M, \end{array}$$

where

$$Y_{i,n+1}:=\left(1-\alpha \right)\left[S_{i,n}+\frac{\left|\Omega \right|}{M}\overset{M}{\underset{j=1}{\sum }}W_{ij}\lambda_{j,n}\right],$$

with

$$\lambda_{j,n}=\{\begin{array}{ll} 1 & \text{if }V_{j,n}>0\\ 0 & \text{if }V_{j,n}<0 \end{array}.$$

Moreover, we have

$$\begin{array}{ll} V_{i,n+1}= & \alpha^{n+1}V_{i,0}+\overset{n}{\underset{k=0}{\sum }}\alpha^{k}Y_{i,n-k}. \end{array}$$

Suppose that for all *n* ∈ ℕ and for all *x* ∈ Ω, *S*_*n*_(*x*) and *W*(*x, y*) are bounded by say *S* and *W*, respectively. Let *Y*_*m*_ = (1 − α)[*S* + |Ω|*W*]. For all 1 ≤ *i* ≤ *M*, for *x*_*i*_ ∈ Ω, and *n* ∈ ℕ we have |*Y*_*i,n*+1_| ≤ *Y*_*m*_, and

$$\begin{array}{lll} \left|V_{i,n+1}\right| & \leq & \alpha^{n}|V_{i,0}|+Y_{m}\cdot \frac{1-\alpha^{n+1}}{1-\alpha }. \end{array}$$

This shows that $\underset{n\to \infty }{\text{lim}}V_{i,n}$ exists and $V\left(x\right)=\underset{n\to \infty }{\text{lim}}V_{n}\left(x\right)$ defines the state of the field overtime. If $\underset{n\to \infty }{\text{lim}}V_{n}\left(x\right)<0$, then the field settles to an inhibitory phase overtime, and if $\underset{n\to \infty }{\text{lim}}V_{n}\left(x\right)>0$, the field settles in an excitatory phase overtime. In general *S*_*i,n*_ = ν+*U*_*i,n*_ where ν is a negative real number referred to as the resting level that ensures that the DNFs produce no output in the absence of external input *U*_*i,n*_. If *U*_*i,n*_ ≡ 0 and *V*_0_(*x*) < 0 for all *x* ∈ Ω, then $V_{i,n+1}=\alpha^{n+1}V_{i,0}+\nu$, since λ_*i,n*−*k*_ = 0 for all *k* = 0, ⋯ , *n* and for all 1 ≤ *i* ≤ *M*. Hence we will have $\underset{n\to \infty }{\text{lim}}V_{i,n}=\nu$, see section 3.5.1 below for an illustration.

### 3.4. Multiple-Layers DDNFs Model

Let 1 ≤ *k, m* ≤ *L*. Consider the DDNFs with *L* layers given above and let *x*_*i*_, *x*_*p*_ on the *k*th layer.

$$\begin{array}{l} V_{i,n+1}^{\left(k\right)}=F\left(V_{i,n}^{\left(k\right)}\right)=\alpha V_{i,n}^{\left(k\right)}+\left(1-\alpha \right)\underset{\ell \in L_{k}}{\sum }\overset{M_{\ell }}{\underset{j=1}{\sum }}\beta_{\ell }\cdot W_{ij}^{\left(k,l\right)}\cdot G\left(V_{j,n}^{\left(\ell \right)}\right)\\ \;+\left(1-\alpha \right)S_{i,n}^{\left(k\right)}. \end{array}$$

Consider a fixed point solution $V_{i,*}^{\left(k\right)}$. Given a layer 1 ≤ *k* ≤ *L* and a neuron at position *x*_*i*_ on it, there are two sources of variability for the potential $V_{i,n+1}^{\left(k\right)}={\left[f_{k}\left(V_{n}\left(X\right)\right)\right]}_{i}$: firstly, the variability due to the *M*_*k*_ neurons on the *k*th layer connected to the neuron at position *x*_*i*_, and secondly, the variability due to the neurons located on possibly two layers (*k* − 1) and (*k* + 1) connected to the *k*th layer. Therefore, let 1 ≤ *m* ≤ *L* and let *x*_*q*_ be a point on the *m*th layer. By the chain rule, we have

$$\frac{\partial {\left[f_{k}\left(V_{n}\left(X\right)\right)\right]}_{i}}{\partial V_{q,n}^{\left(m\right)}}=\frac{\partial {\left[f_{k}\left(V_{n}\left(X\right)\right)\right]}_{i}}{\partial V_{p,n}^{\left(k\right)}}\cdot \frac{\partial V_{p,n}^{\left(k\right)}}{\partial V_{q,n}^{\left(m\right)}}=\Phi_{i,p,n}^{\left(k,k\right)}\cdot \Delta_{p,q,n}^{\left(k,m\right)},$$

where the variability on the *k*th layer is

$$\begin{array}{l} \Phi_{i,p,n}^{\left(k,k\right)}=\alpha \delta_{ip}+\left(1-\alpha \right)\beta_{k}W_{ip}^{\left(k,k\right)}G^{\prime }\left(V_{p,n}^{\left(k\right)}\right). \end{array}$$

From Equation (7), we obtain the variability due to layers *k* − 1 and *k* + 1:

$$\Delta_{p,q,n}^{\left(k,m\right)}=\frac{\partial V_{p,n}^{\left(k\right)}}{\partial V_{q,n}^{\left(m\right)}}=\{\begin{array}{ll} 1 & \text{if }m=k\\ \beta_{m}W_{pq}^{\left(k,m\right)}G^{\prime }\left(V_{q,n}^{\left(m\right)}\right) & \text{if }m\in L_{k}\backslash \left\{k\right\}\\ 0 & \text{if }m\notin L_{k} \end{array}.$$

Let $M=\overset{L}{\underset{\ell =1}{\sum }}M_{\ell }$. We recall that $K_{ij}^{\left(k,\ell \right)}:=W_{ij}^{\left(k,\ell \right)}G^{\prime }\left(V_{j,*}^{\left(\ell \right)}\right)$. Now we consider a *M* × *M* super-adjacency matrix $A=JF\left(V_{*}\left(X\right)\right)={\left(A^{\left(k,m\right)}\right)}_{1\leq k,m\leq L}$, where ***A***^**(*****k,m*****)**^ is a *M*_*k*_ × *M*_*m*_ block matrix defined as $A^{\left(k,m\right)}=\left(a_{ij}^{\left(k,m\right)}\right)$, where $a_{ij}^{\left(k,m\right)}=\Phi_{i,j}^{\left(k,k\right)}\cdot \Delta_{i,j}^{\left(k,m\right)}$ with

(11) $$\begin{array}{l} \Phi_{i,j}^{\left(k,k\right)}=\alpha \delta_{ij}+\left(1-\alpha \right)\beta_{k}W_{ij}^{\left(k,k\right)}G^{\prime }\left(V_{j,*}^{\left(k\right)}\right)=\alpha \delta_{ij}+\left(1-\alpha \right)\beta_{k}K_{ij}^{\left(k,k\right)}, \end{array}$$

and

(12) $$\begin{array}{l} \Delta_{i,j}^{\left(k,m\right)}=\{\begin{array}{ll} 1 & \text{if }m=k\\ \beta_{m}W_{ij}^{\left(k,m\right)}G^{\prime }\left(V_{j,*}^{\left(m\right)}\right)=\beta_{m}K_{ij}^{\left(k,m\right)} & \text{if }m\in L_{k}\backslash \left\{k\right\}\\ 0 & \text{if }m\notin L_{k} \end{array}. \end{array}$$

Consequently, ***A*^(*k,m*)^ = 0** if *m* ∉ *L*_*k*_, where **0** represents the zero matrix. Therefore, the super-adjacency matrix can be written as

$$A=\left(\begin{array}{ccccccc} A^{\left(1,1\right)} & A^{\left(1,2\right)} & 0 & 0 & \cdots & 0 & 0\\ A^{\left(2,1\right)} & A^{\left(2,2\right)} & A^{\left(2,3\right)} & 0 & \cdots & 0 & 0\\ 0 & A^{\left(3,2\right)} & A^{\left(2,3\right)} & A^{\left(3,4\right)} & \cdots & 0 & 0\\ \vdots & \vdots & \vdots & \vdots & \vdots & \vdots & \vdots \\ 0 & 0 & 0 & \cdots & A^{\left(L-1,L-2\right)} & A^{\left(L-1,L-1\right)} & A^{\left(L-1,L\right)}\\ 0 & 0 & 0 & \cdots & 0 & A^{\left(L,L-1\right)} & A^{\left(L,L\right)} \end{array}\right).$$

Clearly, ***A*** is a block tridiagonal matrix that reduces to a block diagonal matrix if the pulse emission rate function *G* is the Heaviside function and to single *M* × *M* diagonal matrix if *L* = 1. There is a rich literature on the subject of tridiagonal matrices and the research is still on-going. There is no close form formula for the eigenvalues of *A* since they are not obvious to calculate, except for some special cases.

#### 3.4.1. Special Case: Single Neuron Multiple-Layers DDNFs

It special case that is worth mentioning since all *L* layers have exactly one neuron and they are interconnected. In fact in this case, *M*_*k*_ = 1, β_*k*_ = |Ω| for 1 ≤ *k* ≤ *L*, and the super adjacency matrix reduces to an *L* × *L* tridiagonal matrix

$$A=\left(\begin{array}{ccccccc} a_{1} & b_{1} & 0 & 0 & \cdots & 0 & 0\\ c_{1} & a_{2} & b_{2} & 0 & \cdots & 0 & 0\\ 0 & c_{2} & a_{3} & b_{2} & \cdots & 0 & 0\\ \vdots & \vdots & \vdots & \vdots & \vdots & \vdots & \vdots \\ 0 & 0 & 0 & \cdots & c_{L-2} & a_{L-1} & b_{L-1}\\ 0 & 0 & 0 & \cdots & 0 & c_{L-1} & a_{L} \end{array}\right),$$

where from Equations (11) and (12), one has

$$\begin{array}{l} \begin{array}{l} a_{k}=\Phi_{11}^{\left(k,k\right)}\cdot \Delta_{11}^{\left(k,k\right)}=\alpha +\left(1-\alpha \right)|\Omega |K^{\left(k,k\right)},\;\text{ for }1\leq k\leq L\\ b_{k}=\Phi_{11}^{\left(k,k\right)}\cdot \Delta_{11}^{\left(k,k+1\right)}=\left[\alpha +\left(1-\alpha \right)|\Omega |K^{\left(k,k\right)}\right]|\Omega |K^{\left(k,k+1\right)},\\ \text{ for }1\leq k\leq L-1\\ c_{k-1}=\Phi_{11}^{\left(k,k\right)}\cdot \Delta_{11}^{\left(k-1,k\right)}=\left[\alpha +\left(1-\alpha \right)|\Omega |K^{\left(k,k\right)}\right]|\Omega |K^{\left(k-1,k\right)},\\ \text{ for }2\leq k\leq L. \end{array} \end{array}$$

We observe that *K*^(*k,k*)^ is the weighted rate of change of the pulse emission function on the *k*th layer, and *K*^(*k,k*+1)^ can be regarded as the transitional weighted rate of change of the pulse emission function from layer *k* to layer *k* + 1. Likewise, *K*^(*k*−1,*k*)^ is the transitional weighted rate of change of the pulse emission function from layer *k* − 1 to layer *k*. From Molinari (2008), the eigenvalues of A are given by the equation

(13) $$\begin{array}{l} \text{det}\left(A-\lambda I_{L}\right)=\text{tr}\left(\overset{2}{\underset{k=L}{\prod }}\left(\begin{array}{cc} a_{k}-\lambda & -b_{k-1}c_{k-1}\\ 1 & -\lambda \end{array}\right)\cdot \left(\begin{array}{cc} a_{1}-\lambda & 0\\ 1 & 0 \end{array}\right)\right)=0. \end{array}$$

Suppose further that *a*_1_ = *a*_2_ = ⋯ = *a*_*L*_ = *a, b*_1_ = *b*_2_ = ⋯ , *b*_*L*−1_ = *b*, and *c*_1_ = *c*_2_ = ⋯*c*_*L*−1_ = *c*. This means that the weighted rates of change of the pulse emission function $K^{\left(k,k\right)}=K_{0}$ for 1 ≤ *k* ≤ *L*, $K^{\left(k-1,k\right)}=K_{1}$ for 1 ≤ *k* ≤ *L* − 1, and $K^{\left(k,k+1\right)}=K_{2}$ for 2 ≤ *k* ≤ *L*. From Kulkarni et al. (1999), the eigenvalues of *A* will given by

$$\lambda_{k}=a-2\sqrt{bc}\cos\left(\frac{\pi k}{L+1}\right),\;1\leq k\leq L.$$

Now for 1 ≤ *k* ≤ *L*, put

$$\mu_{k}=\left[\alpha +\left(1-\alpha \right)|\Omega |K_{0}\right]\left[1-2|\Omega |\sqrt{K_{1}K_{2}}\cos\left(\frac{\pi k}{L+1}\right)\right].$$

We therefore have the following result.

**Theorem 15**. *Suppose we are in the presence of a DDNFs with L layers with M* = 1 *point each, interconnected via a positive connectivity function W and a rate pulse emission rate function G whose rate of change is identical on all layers. Then*

1. *The fixed point **V_∗_*****(*****X*****)** *is asymptotically stable if and only if* $\underset{1\leq k\leq L}{\text{max}}|\mu_{k}|<1$.
2. *The fixed point **V_∗_*****(*****X*****)** *is unstable if and only if* $\underset{1\leq k\leq L}{\text{max}}|\mu_{k}|>1$.
3. *The fixed point **V_∗_*****(*****X*****)** *is maybe stable or unstable if* $\underset{1\leq k\leq L}{\text{max}}|\mu_{k}|=1$.

Proof. By hypothesis, *a* = α + (1 − α)|Ω|*K*_0_, *b* = [α + (1 − α)|Ω|*K*_0_]|Ω|*K*_1_, and *c* = [α + (1 − α)|Ω|*K*_0_]|Ω|*K*_2_, for some *K*_0_, *K*_1_, *K*_2_ > 0. For 1 ≤ *k* ≤ *L*, the eigenvalues are given as

$$\begin{array}{l} \lambda_{k}=a-2\sqrt{bc}\cos\left(\frac{\pi k}{L+1}\right)\\ \;=a-2\sqrt{a^{2}|\Omega {|}^{2}K_{1}K_{2}}\cos\left(\frac{\pi k}{L+1}\right)\\ \;=a\left[1-2|\Omega |\sqrt{K_{1}K_{2}}\cos\left(\frac{\pi k}{L+1}\right)\right]\\ \;=\left[\alpha +\left(1-\alpha \right)|\Omega |K_{0}\right]\left[1-2|\Omega |\sqrt{K_{1}K_{2}}\cos\left(\frac{\pi k}{L+1}\right)\right]\\ \;=\mu_{k}. \end{array}$$

From Thereom 3, the proof is complete.

**Remark 16**.

**Figure 4:**
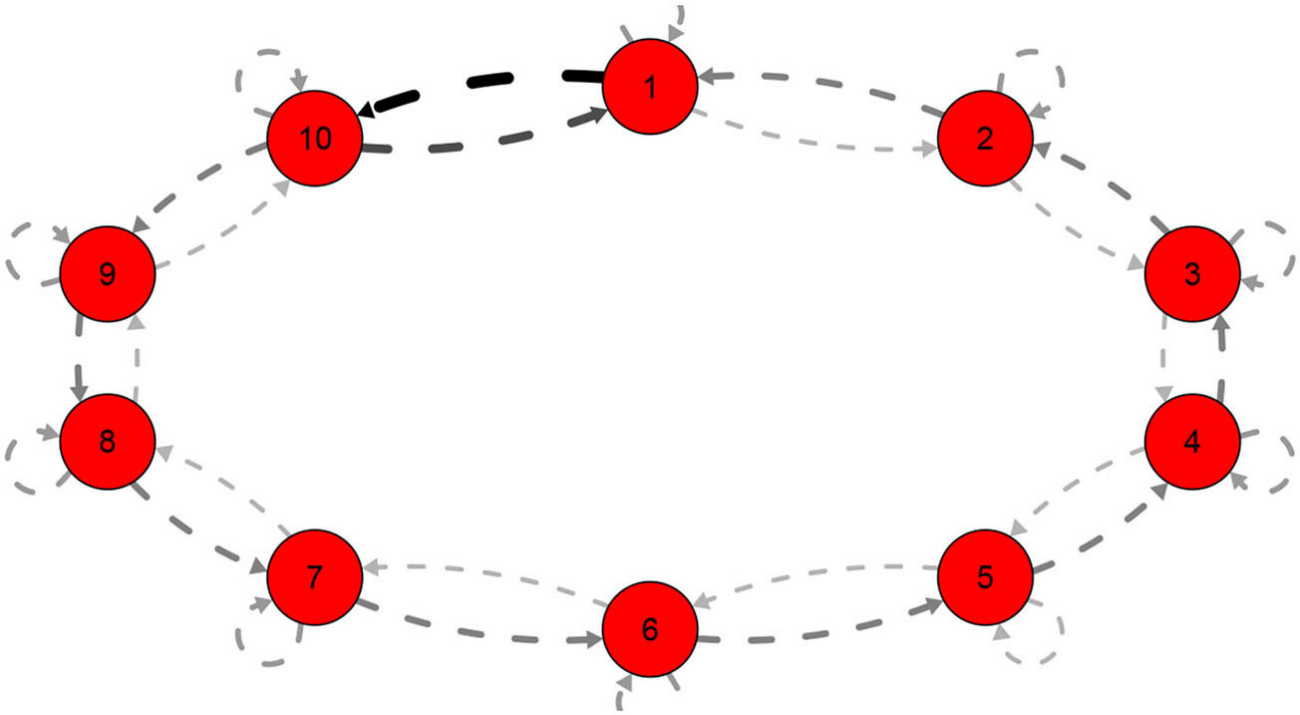
Closed graph with 10 layers for *a* = 4, *b* = 3, *c* = 5, *c*_0_ = 7, *b*_10_ = 10.

1. *It is worth observing that in case L* = 1 and *M* = 1, *then K*_1_ = *K*_2_ = 0 *and thus μ*_1_ = *α* + (1 − *α*)|Ω|*K*_0_. *We see that |μ*_1_ < 1 *if* |Ω|*K*_0_ < 1 *yielding the condition obtained in the one-layer-model*.
2. *We also note that in fact* $\underset{1\leq k\leq L}{\text{max}}|\mu_{k}|=\left[\alpha +\left(1-\alpha \right)|\Omega |K_{0}\right]\left[1+2|\Omega |\sqrt{K_{1}K_{2}}\right]$ *and therefore the stability condition* $\left[\alpha +\left(1-\alpha \right)|\Omega |K_{0}\right]\left[1+2|\Omega |\sqrt{K_{1}K_{2}}\right]<1$ *links all the parameters of system*.
3. *This special cases is also important in that we can imagine a large number of neurons supposedly placed on individual layers and interconnected in a forward or backward manner only and each receiving external input. In case the first and last neurons are connected so that the system forms a weighted closed graph, like in Figure 4, where the thickness represents the weights*.
  *Then the super-adjacency matrix given as*
  $$A=\left(\begin{array}{ccccccc} a_{1} & b_{1} & 0 & 0 & \cdots & 0 & c_{0}\\ c_{1} & a_{2} & b_{2} & 0 & \cdots & 0 & 0\\ 0 & c_{2} & a_{3} & b_{2} & \cdots & 0 & 0\\ \vdots & \vdots & \vdots & \vdots & \vdots & \vdots & \vdots \\ 0 & 0 & 0 & \cdots & c_{L-2} & a_{L-1} & b_{L-1}\\ b_{L} & 0 & 0 & \cdots & 0 & c_{L-1} & a_{L} \end{array}\right).$$
  *According to Molinari (2008), the eigenvalues are given by*
  (14) $$\begin{array}{l} \begin{array}{l} det\left(A-\lambda I_{L}\right)={\left(-1\right)}^{L+1}\left(\overset{L}{\underset{i=1}{\prod }}b_{i}+\overset{L-1}{\underset{i=0}{\prod }}c_{i}\right)\\ \;+tr\left(\overset{2}{\underset{i=L}{\prod }}\left(\begin{array}{cc} a_{i}-\lambda & -b_{i-1}c_{i-1}\\ 1 & -\lambda \end{array}\right)\cdot \left(\begin{array}{cc} a_{1}-\lambda & b_{L}c_{0}\\ 1 & 0 \end{array}\right)\right)\\ \;=0. \end{array} \end{array}$$

#### 3.4.2. Special Case: Single Neuron Two-Layer DDNFs

We now discuss a single neuron two-layer DDNFs. In this case, we still have *M*_*k*_ = 1 for *k* = 1, 2 and *L* = 2. It follows that the super-adjacency matrix is given as $A=\left(\begin{array}{cc} a_{1} & b_{1}\\ c_{1} & a_{2} \end{array}\right)$ where from Equations (11) and (12), we have

$$\begin{array}{l} a_{1}=\alpha +\left(1-\alpha \right)|\Omega |K^{\left(1,1\right)},\\ b_{1}=\left[\alpha +\left(1-\alpha \right)|\Omega |K^{\left(1,1\right)}\right]|\Omega |K^{\left(1,2\right)},\\ a_{2}=\alpha +\left(1-\alpha \right)|\Omega |K^{\left(2,2\right)},\\ c_{1}=\left[\alpha +\left(1-\alpha \right)|\Omega |K^{\left(2,2\right)}\right]|\Omega |K^{\left(2,1\right)}. \end{array}$$

Let

$$\begin{array}{l} \theta =1-|\Omega {|}^{2}K^{\left(1,2\right)}K^{\left(2,1\right)},\\ \mu_{0}=|\Omega |\left[K^{\left(1,1\right)}+K^{\left(2,2\right)}\right],\\ \mu =\left[\alpha \left(K^{\left(1,1\right)}+K^{\left(2,2\right)}\right)+\left(1-\alpha \right)|\Omega |K^{\left(1,1\right)}K^{\left(2,2\right)}\right]. \end{array}$$

We have the following stability result.

**Theorem 17**. *Consider a two-layer DDNFs with one neuron each. Then the nontrivial solution **V_∗_(X)** is asymptotically stable if and if the following conditions are satisfied:*

1. 0 < θ < 1,
2. μ < 1,
3. ${\left(\mu \theta +2-\mu_{0}\right)}^{2}<4\theta \left(\mu \theta +1-\mu_{0}\right)$.

Proof. By the determinant-trace analysis, see for instance Elaydi (2007), it is enough to prove that the above conditions are equivalent to

|tr(A)|-1<det(A)<1.

The determinant of the super-adjacency matrix is

$$\begin{array}{l} \text{det}\left(A\right)=a_{1}a_{2}-b_{1}c_{1}\\ \;=a_{1}a_{2}\left[1-|\Omega {|}^{2}K^{\left(1,2\right)}K^{\left(2,1\right)}\right]\\ \;=[\alpha^{2}+\alpha \left(1-\alpha \right)|\Omega |\left(K^{\left(1,1\right)}+K^{\left(2,2\right)}\right)\\ \;+{\left(\left(1-\alpha \right)|\Omega |\right)}^{2}K^{\left(1,1\right)}K^{\left(2,2\right)}]\left[1-|\Omega {|}^{2}K^{\left(1,2\right)}K^{\left(2,1\right)}\right]\\ \;=[\alpha^{2}+\left(1-\alpha \right)|\Omega |[\alpha \left(K^{\left(1,1\right)}+K^{\left(2,2\right)}\right)\\ \;+\left(1-\alpha \right)|\Omega |K^{\left(1,1\right)}K^{\left(2,2\right)}]]\left[1-|\Omega {|}^{2}K^{\left(1,2\right)}K^{\left(2,1\right)}\right]\\ \;=\left[\alpha^{2}+\left(1-\alpha \right)\mu \right]\theta . \end{array}$$

Also

$$\begin{array}{l} \text{tr}\left(A\right)=|\text{tr}\left(A\right)|=a_{1}+a_{2}=2\alpha +\left(1-\alpha \right)|\Omega |\left[K^{\left(1,1\right)}+K^{\left(2,2\right)}\right]\\ \;=2\alpha +\left(1-\alpha \right)\mu_{0}. \end{array}$$

Therefore since θ < 1 and μ < 1, we have that det(*A*) < 1. Let $P\left(\alpha \right):=\text{det}\left(A\right)-\text{tr}\left(A\right)+1=\theta \alpha^{2}+\left(\mu \theta +2-\mu_{0}\right)\alpha +\mu \theta +1-\mu_{0}$. The discriminant of *P*(α) is $\Delta ={\left(\mu \theta +2-\mu_{0}\right)}^{2}-4\theta \left(\mu \theta +1-\mu \right)$. Therefore *P*(α) is positive if Δ < 0 with θ > 0, that is, ${\left(\mu \theta +2-\mu_{0}\right)}^{2}-4\theta \left(\mu \theta +1-\mu \right)$ with θ > 0.

**Remark 18**.

1. *We note that the inequality θ < 1 must be strict, otherwise the third condition in the theorem would be false. To see why, observe that θ* = 1 ⇒ *L* = 1 *thus K*^(2,2)^ = 0. *Hence* $\mu_{0}=|\Omega |K^{\left(1,1\right)}$ *and* μ = α*K*^(1,1)^. *Therefore*, ${\left(\mu \theta +2-\mu_{0}\right)}^{2}<4\theta \left(\mu \theta +1-\mu_{0}\right)\Leftrightarrow {\left(\alpha K^{\left(1,1\right)}+2-|\Omega |K^{\left(1,1\right)}\right)}^{2}<4\left(\alpha K^{\left(1,1\right)}+1-|\Omega |K^{\left(1,1\right)}\right)$, *that is*, ${\left((\alpha -|\Omega |)K^{\left(1,1\right)}+2\right)}^{2}<4(\alpha -|\Omega |)K^{\left(1,1\right)}+1)$ *which is impossible since the latter would be equivalent to saying* ((α − |Ω|)*K*^(1,1)^)^2^ < 0.
2. *We observe that the condition* 0 < θ < 1 *requires* |Ω|^2^*K*^(1,2)^*K*^(2,1)^ < 1. *What is striking about it is that is achieved if* $\max\left\{K^{\left(1,2\right)},K^{\left(2,1\right)}\right\}<\frac{1}{\left|\Omega \right|}$, *similarly to Remark 14 above*.

### 3.5. Simulations

**Figure 5:**
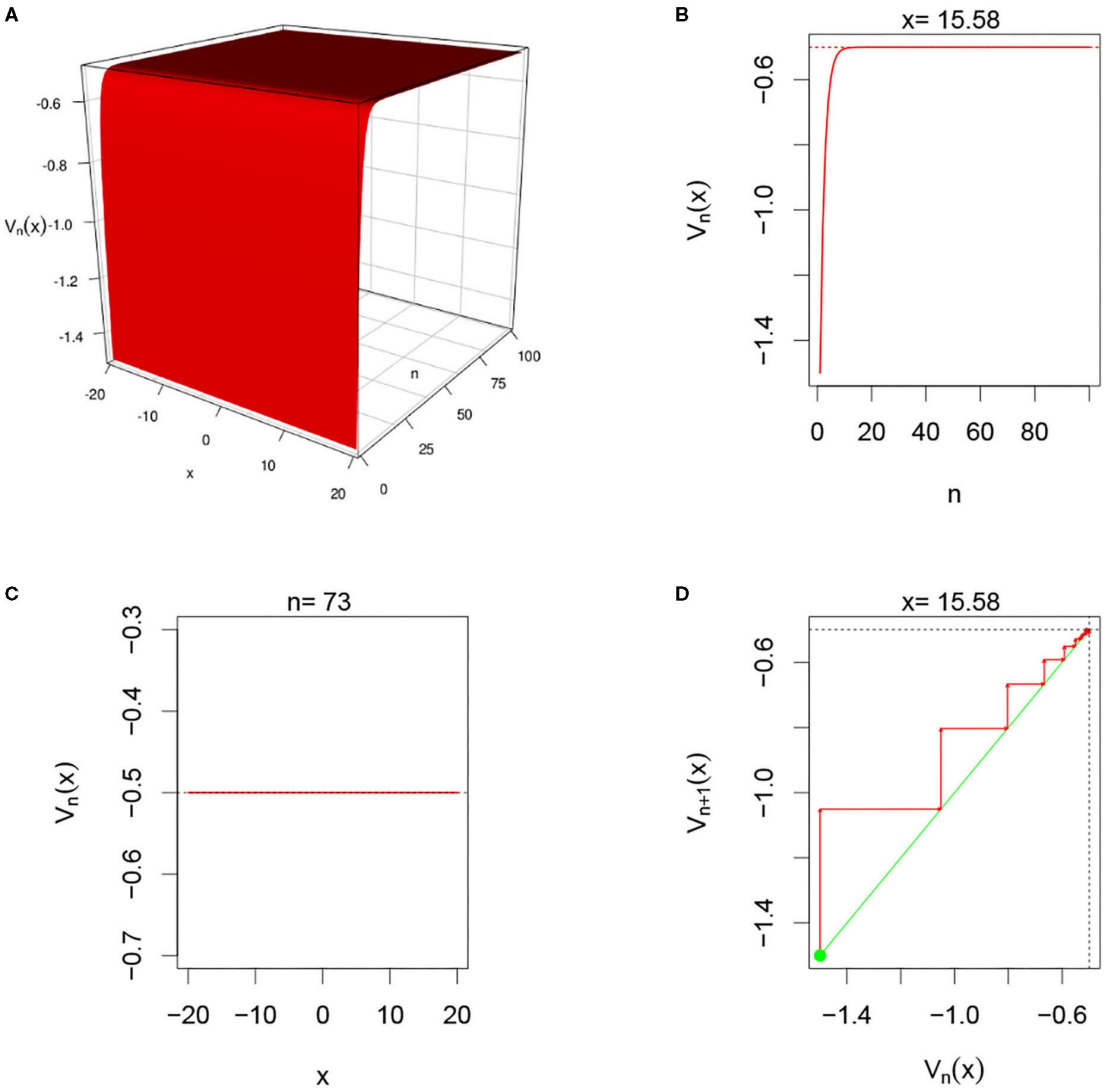
Here *U*_*i,n*_ = 0 and *G*(*v*) = *G*_1_(*v*). **(A–D)** We observe from all graphs that *V*_*n*_(*x*) quickly settles to the resting level ν = −0.5 confirming the theoretical results in section 3.3.2.

**Figure 6:**
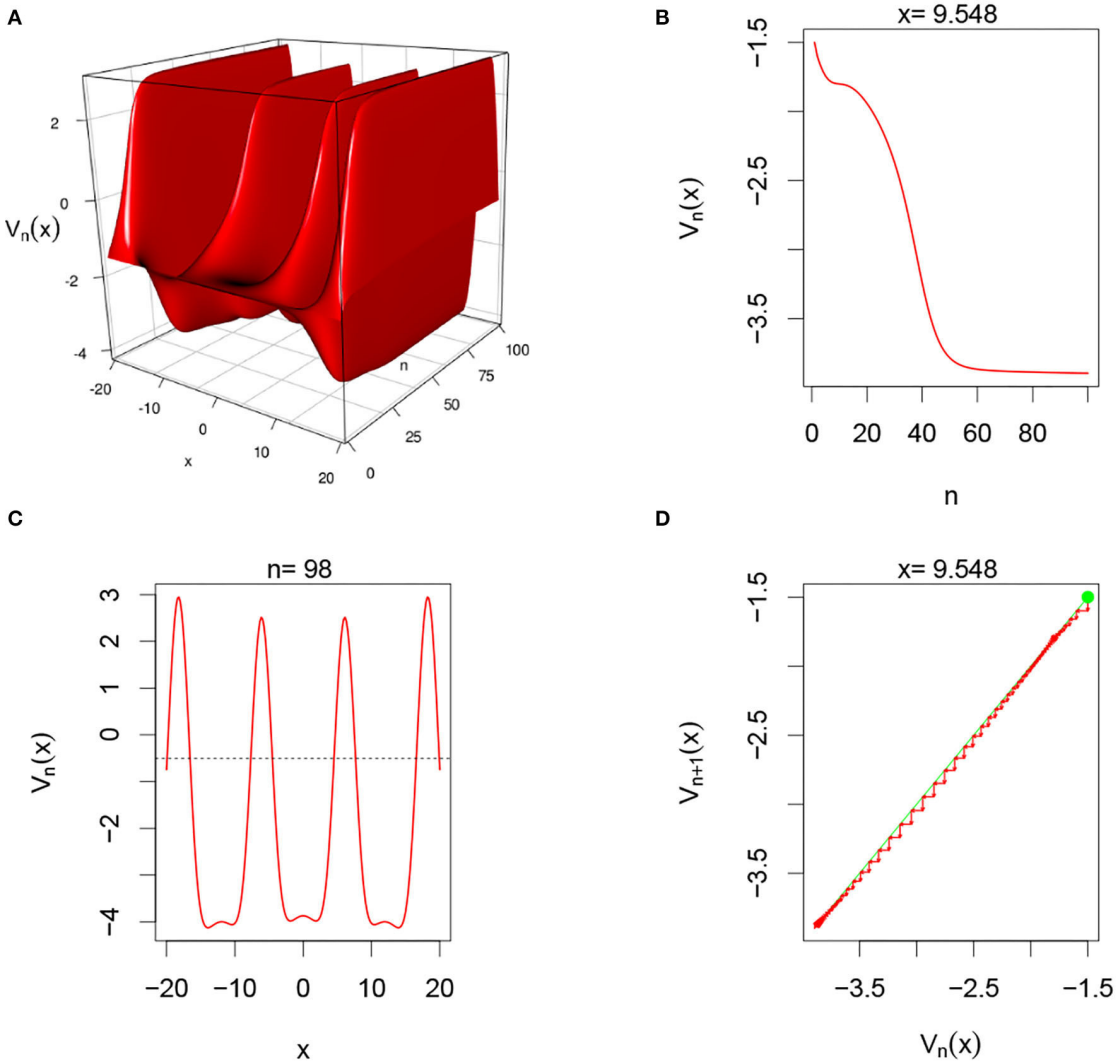
Here *U*_*i,n*_ = 0 and *G*(*v*) = *G*_2_(*v*). From **(A)**, we observe that system alternating between inhibitory and excitatory phases. **(B,D)** Show that for fixed *x* = 9.548, the system converges overtime. In **(C)**, we clearly see the oscillatory regime of the system in the space domain for fixed *n* = 98.

**Figure 7:**
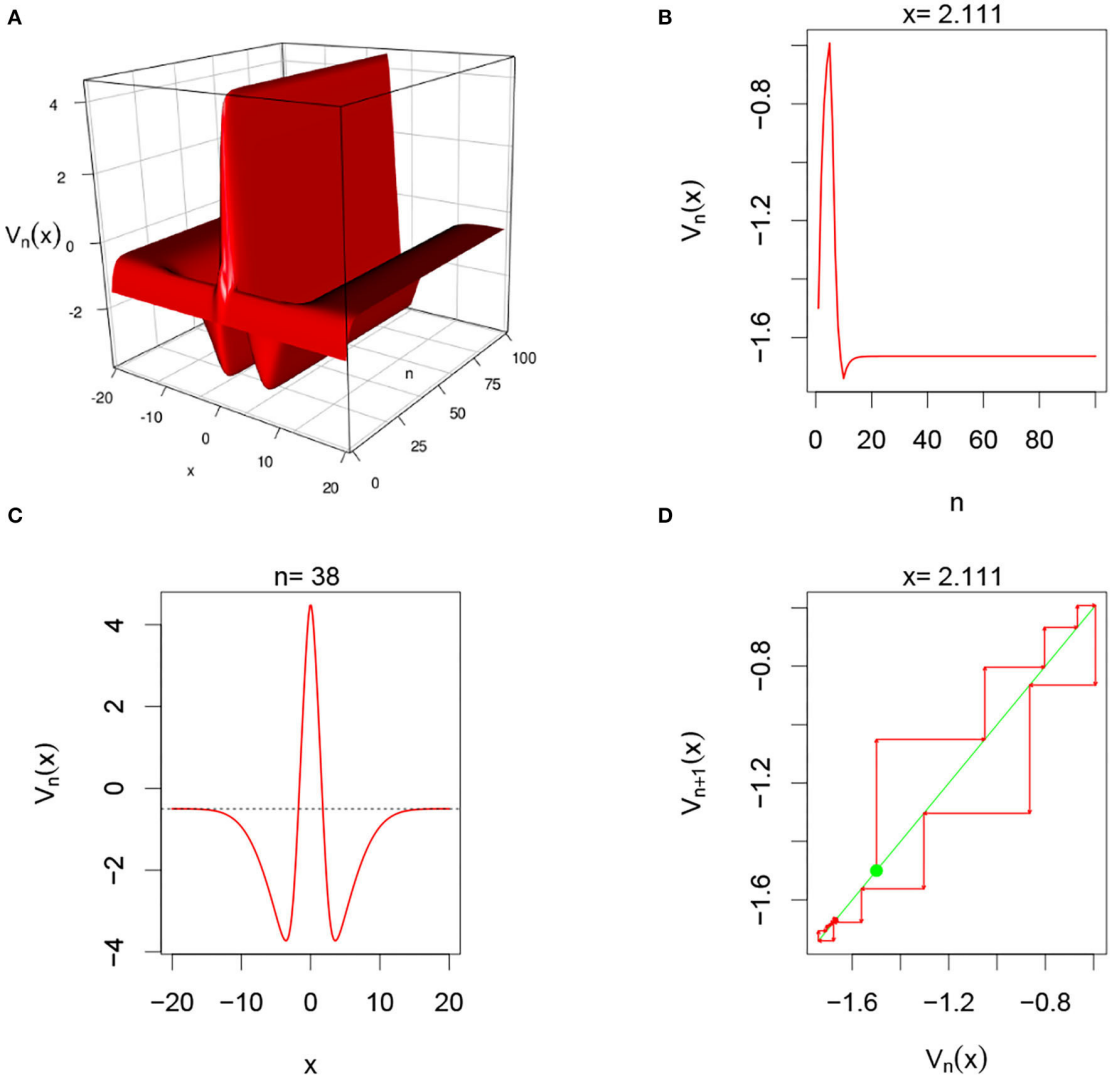
Here $U_{i,n}={\left(2\pi \right)}^{-1/2}e^{-\frac{1}{2}\cdot x_{i}^{2}}$ and *G*(*v*) = *G*_1_(*v*). From **(A)**, we observe that system has an excitatory phase around 0 and an inhibitory phase farther away. **(B,D)** Show that for fixed *x* = 2.111, the system converges overtime. In **(C)**, we clearly observe a high level excitation occurring around 0, and the system settling back to its resting phase farther away from 0 for fixed *n* = 38.

**Figure 8:**
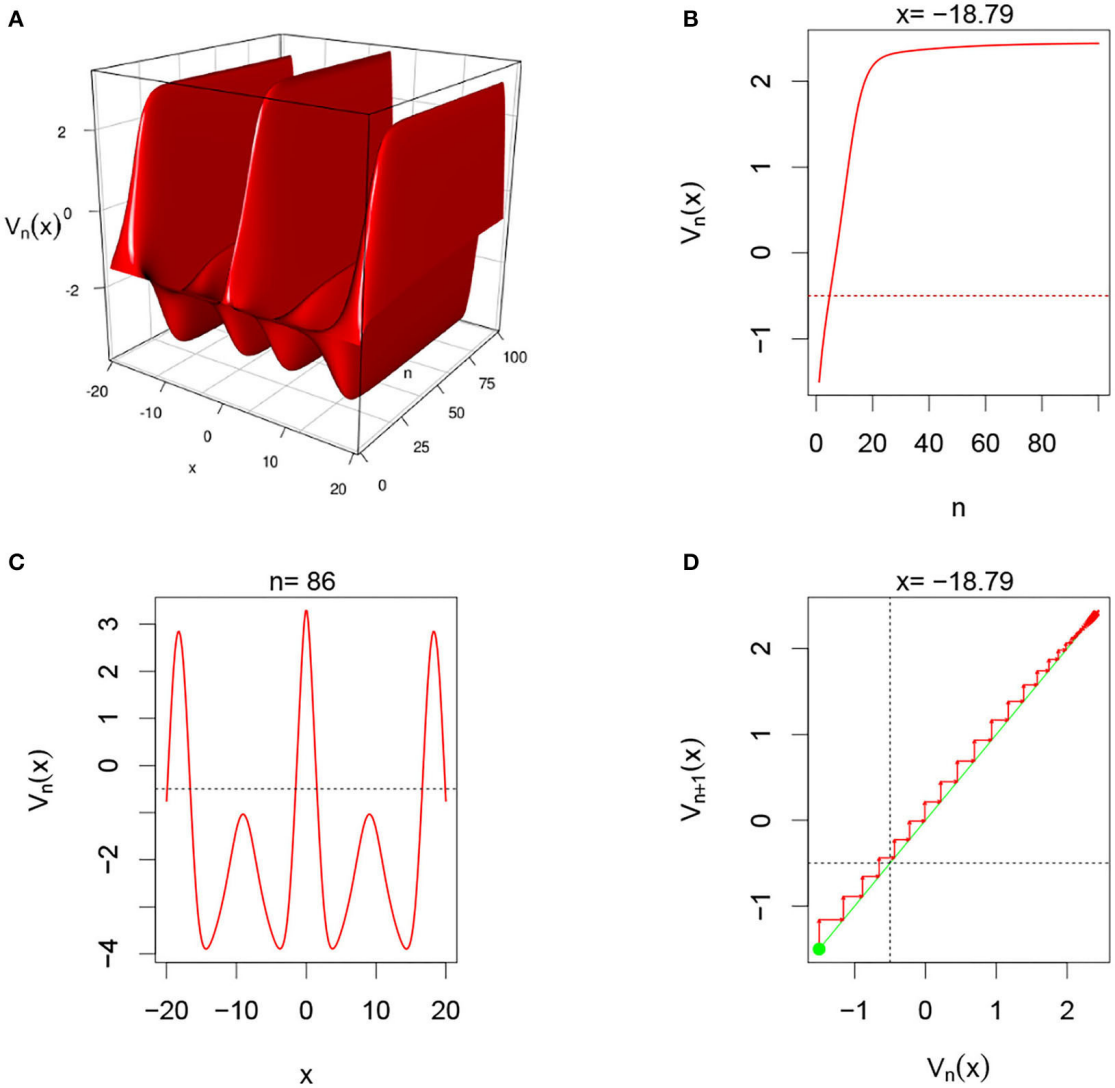
Here $U_{i,n}={\left(2\pi \right)}^{-1/2}e^{-\frac{x_{i}^{2}}{2}}$ and *G*(*v*) = *G*_2_(*v*). This case is similar to the second case above. From **(A)**, we observe that system also alternates between inhibitory and excitatory phases. **(B,D)** Show that for fixed *x* = −18.79, the system converges overtime. In **(C)**, we clearly the oscillatory regime of the system in the space domain for fixed *n* = 86. The system in this case is more frequently inhibitory than excitatory.

There are multiple parameters that affect the stability of a fixed point solution to the DDNFs: the choice of the kernel function *W*(*x, y*), the pulse emission rate function *G*, parameters such the size of the field |Ω|, the time scale function ϕ(*h*), and the number of points per layer *M*, and the length of time *N*. In these simulations, we will consider the Ω = [−20, 20], *h* = 0.8, *N* = 100, *M* = 200, and the $W\left(x,y\right)=\sigma^{+}e^{-0.5{\left(x-y\right)}^{2}/\sigma_{1}^{2}}-\sigma^{-}e^{-0.5{\left(x-y\right)}^{2}/\sigma_{2}^{2}}$, with $\sigma^{+}=4,\sigma^{-}=1.5,\sigma_{1}=1$, and σ_2_ = 4.5. We select a resting phase ν = −0.5. To initialize our dynamical system, we will choose *V*_*i*, 0_ = −1.5 for *i* = 1, 2, ⋯ , *M*. In Figures 5–**8**, below, (a) is the three dimension representation of *V*_*n*_(*x*): = *V*(*x, n*), (b) represents *V*_*n*_(*x*) as a function of *n* for a given *x* value, (c) represents *V*_*n*_(*x*) as function of *x* for a given *n* value, and (*d*) represents the cobweb diagram for a given *x* value, namely a plot of *V*_*n*+1_(*x*) vs. *V*_*n*_(*x*) for which the green dot represents the starting point *V*_0_(*x*) = −1.5 and the red arrows connecting the points with coordinates [*V*_*n*_(*x*), *V*_*n*_(*x*)] and [*V*_*n*_(*x*), *V*_*n*+1_(*x*)] to show the evolution of the dynamical system overtime. Convergence occurs when the arrows stop evolving, and oscillations occurs when they circle around indefinitely. In the multi-layer case, we let *L* = 250.

#### 3.5.1. Single-Layer With Heaviside Function and No External Input

In this simulation, we consider single-layer DDNFs with *U*_*i,n*_ = 0 with a Heaviside pulse emission function, see Figure 5 below.

#### 3.5.2. Single-Layer With Sigmoid Function and No External Input

In this simulation, we consider single-layer DDNFs with *U*_*i,n*_ = 0 but with a sigmoid pulse emission function, see Figure 6 below.

#### 3.5.3. Single-Layer With Heaviside Function With Gaussian External Input

In this simulation, we consider single-layer DDNFs with a Heaviside Pulse emission function with a Gaussian external input $U_{i,n}={\left(2\pi \right)}^{-1}e^{\frac{1}{2}x_{i}^{2}}$, see Figure 7 below.

#### 3.5.4. Single-Layer With Sigmoid Function With Gaussian External Input

In this simulation, we consider single-layer DDNFs with a Sigmoid Pulse emission function with a Gaussian external input $U_{i,n}={\left(2\pi \right)}^{-1}e^{\frac{1}{2}x_{i}^{2}}$, see Figure 8 below.

#### 3.5.5. Multiple-Layer With Sigmoid Function With No External Input

**Figure 9:**
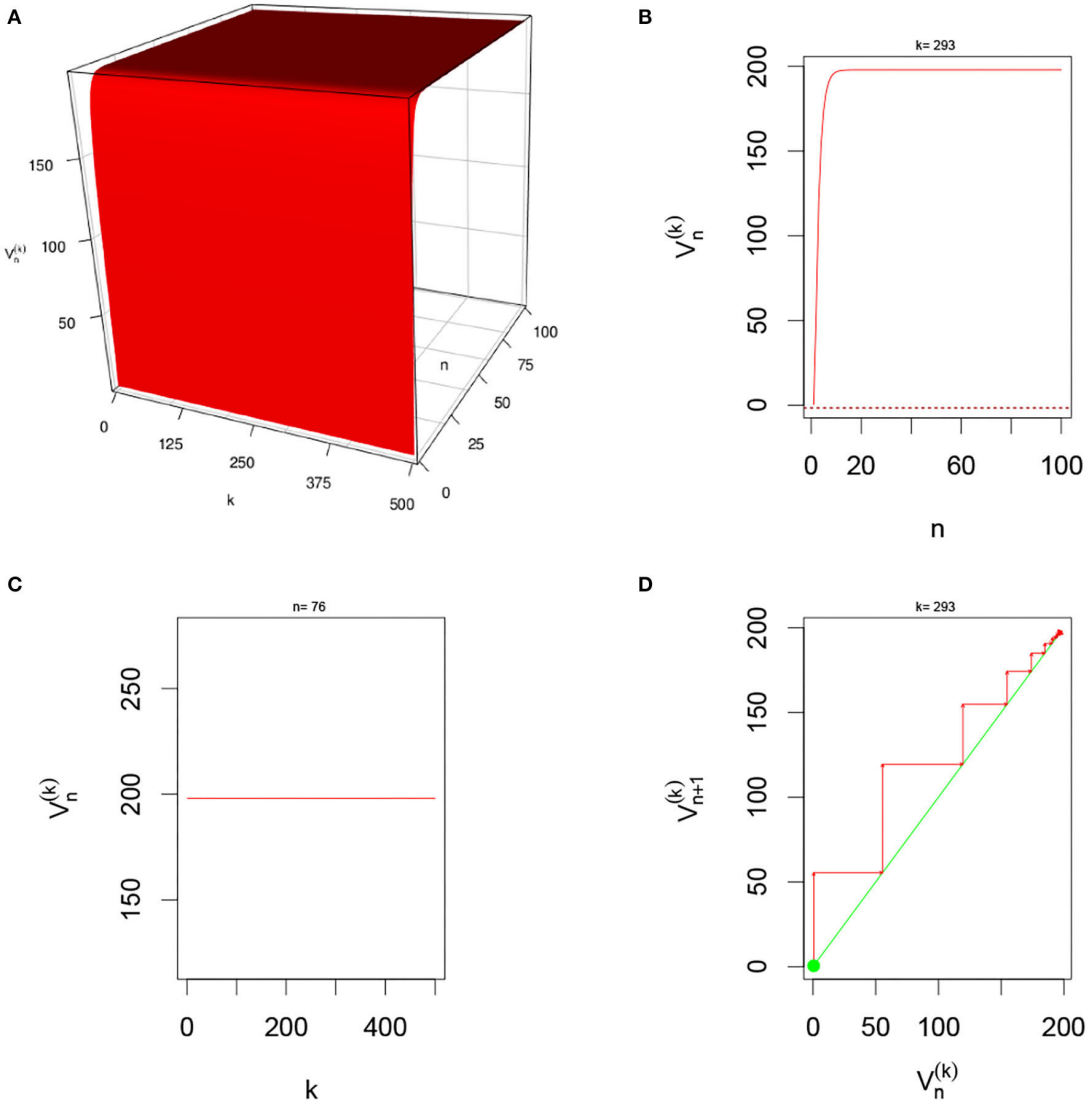
Here *U*_*i,n*_ = 0 and *G*(*v*) = *G*_2_(*v*) with *L* = 200 and *M* = 1 neuron per layer. From **(A)**, we observe that system quickly excitatory and settles into constant state overtime. **(B,D)** Show that for fixed *k* = 293, the system converges overtime. In **(C)**, we clearly see the constant state for *n* = 76.

In this simulation, we consider Multiple-layer DDNFs with a Sigmoid pulse emission rate function with no external input, *U*_*i,n*_ = 0, see Figure 9 below.

#### 3.5.6. Multiple-Layer With Sigmoid Function With Gaussian External Input

**Figure 10:**
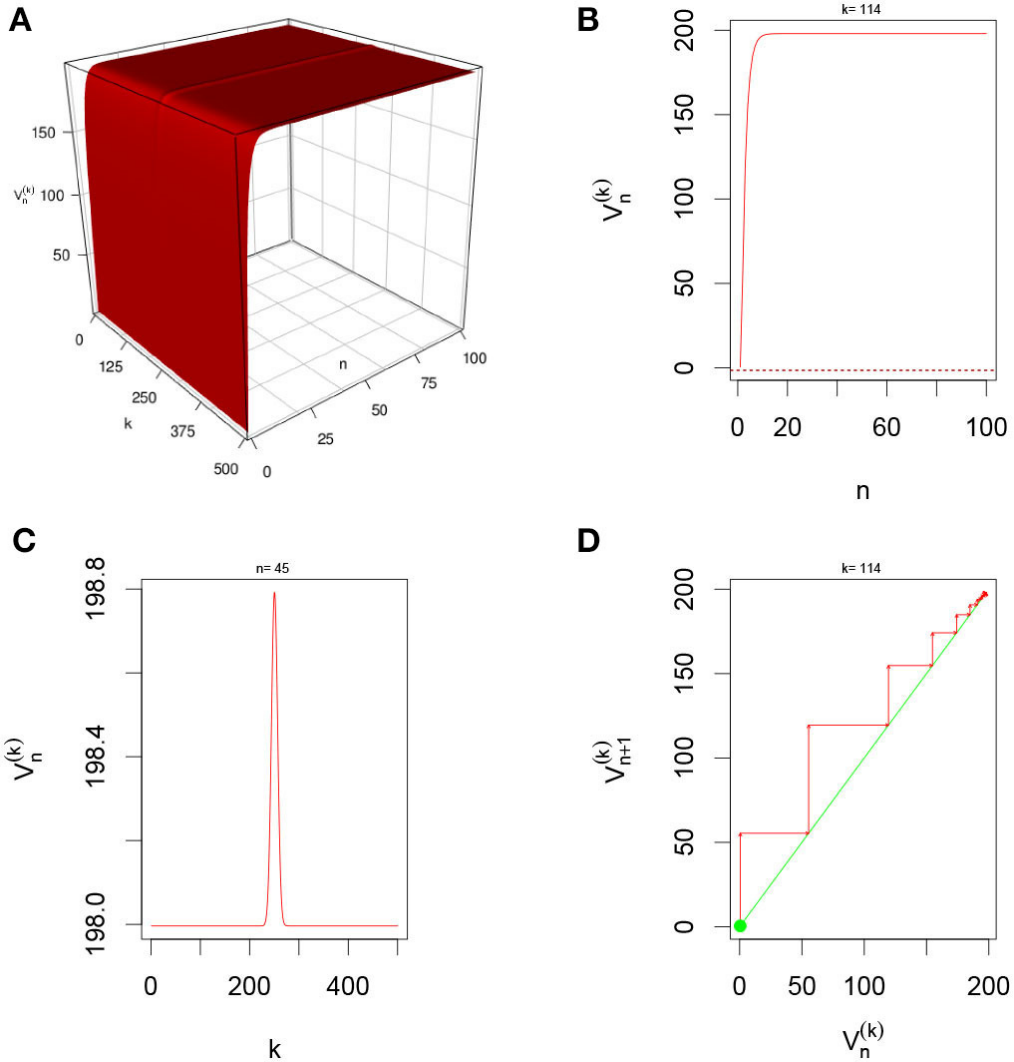
Here $U_{i,n}={\left(2\pi \right)}^{-1/2}e^{-\frac{x_{i}^{2}}{2}}$and *G*(*v*) = *G*_2_(*v*) with *L* = 200 and *M* = 1 neuron per layer. From **(A)**, we observe that system is quickly excitatory and settles into constant “Gaussian” state overtime. **(B,D)** Show that for fixed *k* = 114, the system converges overtime. In **(C)**, we clearly see the “Gaussian” state for *n* = 45 with peaks located at around *L* = 250.

In this simulation, we consider Multiple-layer DDNFs with a Sigmoid pulse emission rate function with a Gaussian external input, *U*_*i,n*_ = 0, see Figure 10 below.

#### 3.5.7. Effect of α

**Figure 11:**
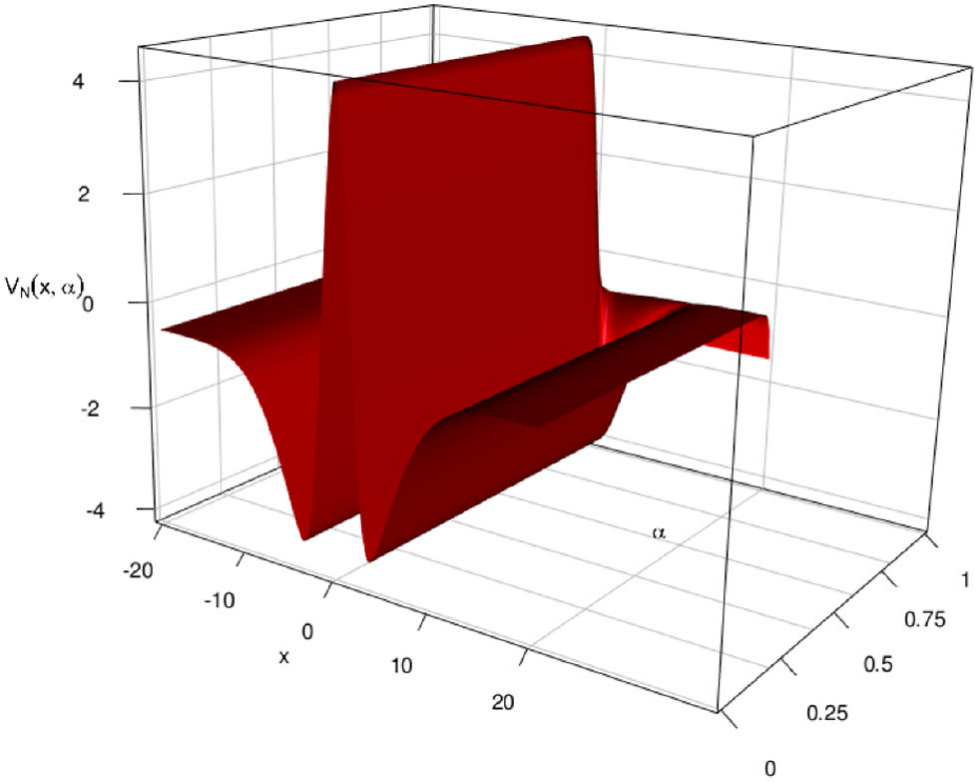
Here $U_{i,n}={\left(2\pi \right)}^{-1/2}e^{-\frac{x_{i}^{2}}{2}}$ and *G*(*v*) = *G*_2_(*v*) with *L* = 1 and *M* = 200 neuron per layer. We see that the cross-sections are as in Figure 7 above and the “flaps” at the end represent the case where α = 1, which amounts to the constant case.

In this simulation, we consider *V*_*N*_(*x*, α) where *N* = 100 for different values of α. Since the convergence to the fixed solution is quick, we expect *V*_*N*_(*x*, α) to be independent of α at *n* = *N* = 100, in that any cross-section of the three dimensional plot *V*_*N*_(*x*, α) should be the same. This is shown in Figure 11 below for a *G*(*v*) = *G*_1_(*v*) and $U_{i,n}={\left(2\pi \right)}^{-1/2}e^{-\frac{x_{i}^{2}}{2}}$.

## 4. Discussion

In this paper, we have proposed a discrete model for dynamical neural fields. We have proposed another proof of the existence of nontrivial solutions for dynamic neural fields. We have discussed its stability of nontrivial solutions of dynamic neural field for some particular cases. The simulations we propose capture very well the notion of stability on dynamic neural field. Below are some observations drawn from this analysis.

1. We observe that the cases of no external input *U*_*i,n*_ with one layer and multiple neurons and multiple layers with one neuron each are very similar, albeit the shape of the potential function $V_{i,n}^{\left(k\right)}$. In fact, this is not surprising since multiple layers with one neurons each amount to one layer with neurons connected like in a graph.
2. An important observation is that of the stability conditions depend on the knowledge of a nontrivial solution *V*_∗_ for the DDNFs. Two things to note about ***V*_∗_(*X*)** are:
  - We can not obtain ***V*_∗_(*X*)** in a close form. We may however rely on estimates like the one proposed in Kwessi (2021). Even in the latter case, using an estimate may proved delicate because of limited accuracy.
  - If one cannot use a true value for ***V*_∗_(*X*)**, one may use the properties of the pulse emission functions such as the Heaviside or sigmoid functions that are in most cases in applications bounded.
3. We note the size of the domain Ω is an important parameter for the shape of $V_{i,n}^{\left(k\right)}$.
4. As for the types of kernels, we note that Gaussian and Laplacian kernel tends to produce similar results that are sometimes very different from those produced by hyperbolic tangent kernels.
5. We note that the choice of α = *e*^−*h*^ is similar to an Euler forward discretization with $e^{-h}=\frac{dt}{\tau }$ where *dt* ≪ τ. This choice is made for simplification purposes and also out of an abundance of caution due to the fact for certain one-dimension systems, the Euler forward discretization have been shown not to always preserve the true dynamics from the ordinary differential equation, see for instance Kwessi et al. (2018).
6. Another important note is that the parameter α = 1 was not found to be a bifurcation parameter, thus the bifurcation diagram will not be given for sake of simplicity.
7. We acknowledge that in the two special cases discussed for multiple-layers DDNFs, so far, at least theoretically, the results apply only to positive connectivity functions *W*(*x, y*) because we rely on results previously established in the current literature. Simulations however suggest that they may also extend to negative connectivity functions and it would a worthwhile exercise to see how to establish these results theoretically.

## Data Availability Statement

The original contributions presented in the study are included in the article/supplementary material, further inquiries can be directed to the corresponding author/s.

## Author Contributions

The author confirms being the sole contributor of this work and has approved it for publication.

## Conflict of Interest

The author declares that the research was conducted in the absence of any commercial or financial relationships that could be construed as a potential conflict of interest.
